# CuO@Pyridine Composite for Efficient Removal of Malachite Green and Cd(II) from Water: Adsorption Performance and Mechanistic Insights

**DOI:** 10.3390/molecules31091501

**Published:** 2026-04-30

**Authors:** Marwa M. Abdeen, Mohamed G. Abouelenein, Marwa Abd Elfattah, Safinaz H. El-Demerdash, Marwa A. Abdelhameed, Sara M. Elnagar, Mariam T. Yasin, Donia F. Elhadad, Mohamed Mostafa A. Mohamed

**Affiliations:** 1 Basic Science Department, Higher Institute of Engineering and Technology, El-Bagour, Menoufia, Egypt; marwamaherabdeen94@gmail.com; 2 Chemistry Department, Faculty of Science, Menoufia University, Shebin El-Koam P.O. Box 32511, Menoufia, Egypt; drmoh.gamal@science.menofia.edu.eg (M.G.A.); hamdysafinaz@yahoo.com (S.H.E.-D.); marwa.abdalhameed1701168@science.menofia.edu.eg (M.A.A.); sara27mohamed2@gmail.com (S.M.E.); mariamtamer663@gmail.com (M.T.Y.); doniaf134@gmail.com (D.F.E.); 3 Chemical Engineering Department, Higher Institute of Engineering and Technology, El-Bagour, Menoufia, Egypt; mzmi2005@gmail.com; 4 Civil and Environmental Engineering Department, United Arab Emirates University, Al-Ain P.O. Box 15551, United Arab Emirates

**Keywords:** adsorption, pyridine, nano-CuO, malachite green, Cd(II), wastewater treatment

## Abstract

A heteroatom-rich pyridine-based adsorbent (**Pyridine PC**) was synthesized through a multicomponent strategy and structurally confirmed by ^1^H/^13^C NMR spectroscopy and mass spectrometry. To further enhance adsorption activity and surface reactivity, waste-derived CuO nanoparticles were immobilized onto the porous heterocyclic framework, generating a sustainable **CuO@Pyridine PC** hybrid nanocomposite. Batch adsorption experiments demonstrate highly efficient removal of malachite green (MG) dye and Cd(II) ions from aqueous solutions. Kinetic analysis reveals that adsorption follows the pseudo-second-order model, while equilibrium data are best described by the Freundlich isotherm, indicating adsorption on heterogeneous surfaces. Thermodynamic parameters confirm that the adsorption processes are spontaneous and exothermic. Surface and structural characterization using SEM/EDX, elemental mapping analysis and FT-IR before and after adsorption verifies strong pollutant binding and highlights the role of nitrogen- and oxygen-containing functional groups as dominant interaction sites. BET measurements show that CuO incorporation increases surface area and pore volume, while zeta potential analysis indicates excellent colloidal stability of the composite in aqueous media. Consequently, the CuO-modified sorbent exhibits enhanced adsorption capacities, increasing from 169.8 to 176.13 mg g^−1^ for MG and from 276.5 to 368 mg g^−1^ for Cd(II). The adsorbent demonstrated effective pollutant removal from real wastewater. The adsorption mechanism involves synergistic interactions between functional groups in the Pyridine PC matrix and CuO nanoparticles, providing enhanced active binding sites.

## 1. Introduction

The escalating global demand for a clean and sustainable environment has positioned water contamination among the most pressing environmental challenges of the modern era [1]. Rapid industrialization, population growth, and intensive agricultural activities have collectively led to the discharge of large volumes of wastewater containing complex mixtures of organic, inorganic, and microbial pollutants. Among these contaminants, synthetic dyes and heavy metals represent particularly serious threats because of their high toxicity, environmental persistence, and strong tendency for bioaccumulation within aquatic ecosystems [2,3,4,5].

Malachite green (MG), a cationic triphenylmethane dye, is extensively utilized in textile dyeing, paper printing, leather processing, and aquaculture, where it is applied as a fungicide and disinfectant. Despite its widespread industrial use, MG and its reduced metabolite, leuco-malachite green (LMG), exhibit high toxicity and remarkable resistance to biodegradation [6,7]. Even at concentrations below 1 mg L^−1^, these compounds have been reported to induce mutagenic, carcinogenic, and teratogenic effects, posing severe threats to aquatic organisms and human health. Consequently, the effective removal of MG from industrial effluents prior to environmental discharge is essential to minimize ecological damage and public health risks [8,9].

In addition to organic dyes, heavy metals constitute another major class of persistent water pollutants. Unlike many organic contaminants, heavy metals are non-biodegradable and can accumulate in biological tissues, leading to long-term toxicity and biomagnification within food chains [10,11]. Among them, cadmium (Cd^2+^) is particularly hazardous and has been classified as a human carcinogen, with well-documented adverse effects on renal, hepatic, and pulmonary systems. Cadmium contamination primarily originates from industrial activities such as electroplating, pigment production, battery manufacturing, and metal refining processes. Owing to its high solubility and mobility in aqueous environments, Cd^2+^ is difficult to remove efficiently, thereby necessitating the development of effective and sustainable remediation strategies [12,13,14].

Various physical, chemical, and biological technologies have been developed for the removal of dyes and heavy metals from wastewater, including advanced oxidation processes, membrane separation, ion exchange, coagulation–flocculation, and photocatalysis. Although many of these approaches can achieve high removal efficiencies under control conditions, their practical implementation is often constrained by high operational costs, complex maintenance requirements, and the generation of secondary pollutants. For example, advanced oxidation processes require significant energy input, coagulation processes generate large volumes of sludge requiring further treatment, and membrane systems frequently suffer from fouling and high replacement costs. In contrast, adsorption has emerged as a particularly attractive water treatment strategy due to its operational simplicity, high removal efficiency, economic feasibility, and the possibility of adsorbent regeneration [15,16,17].

Activated carbon is widely recognized as a benchmark adsorbent in water treatment because of its large surface area and well-established performance. However, depending on operational scale and regeneration requirements, its production and lifecycle costs may influence its economic feasibility in certain large-scale or cost-sensitive applications. Consequently, considerable research attention has been directed toward developing alternative adsorbent materials that combine high adsorption performance with structural tunability and cost-effective synthesis. Among the proposed material platforms, heterocyclic frameworks have attracted increasing interest due to their electronically rich structures and the presence of heteroatoms such as nitrogen and oxygen, which can function as coordination centers for metal ions and interaction sites for organic contaminants. In particular, pyridine-based systems have emerged as promising candidates because the nitrogen atom within the aromatic ring provides a strong coordination site for metal binding, while the conjugated π-electron system facilitates π–π interactions with aromatic dye molecules, enabling multiple adsorption pathways within a single framework [18,19,20].

Despite these advances, several challenges remain in the design of heterocyclic adsorbents for water purification. Many previously reported materials suffer from limited densities of accessible coordination sites, insufficient structural adaptability for simultaneous adsorption of different classes of pollutants, or multistep synthetic procedures that limit practical scalability. Therefore, the development of structurally tunable heterocyclic adsorbents capable of providing multiple interaction mechanisms while maintaining synthetic simplicity remains an important research objective [21].

In this context, a pyridine-based heterocyclic adsorbent (**Pyridine PC**) was designed and synthesized for the simultaneous removal of malachite green (MG) dye and Cd^2+^ ions from aqueous solutions. The molecular architecture of **Pyridine PC** contains multiple nitrogen- and oxygen-containing functional groups, including amino, cyano, carbonyl, and nitro moieties, which collectively provide abundant active sites for coordination, electrostatic attraction, and hydrogen bonding interactions. In addition, the conjugated aromatic structure of the pyridine framework promotes strong π–π interactions with aromatic dye molecules, thereby enhancing adsorption efficiency toward organic contaminants [22,23].

To further improve the adsorption performance and surface reactivity of the system, copper oxide (CuO) nanoparticles derived from solid waste were immobilized onto the **Pyridine PC** matrix to form a **CuO@Pyridine PC** nanocomposite. Comprehensive physicochemical characterization confirmed the successful formation of the hybrid material. FT-IR, ^1^H NMR, and ^13^C NMR analyses verified the structural integrity of the organic framework, while SEM and EDX analyses demonstrated the effective incorporation of CuO within the adsorbent matrix. Brunauer–Emmett–Teller surface analysis revealed a significant increase in surface area and pore volume following CuO integration, resulting in a higher density of accessible adsorption sites. Additionally, zeta potential measurements confirmed the excellent colloidal stability of the composite in aqueous media, indicating its suitability for water treatment applications.

The adsorption performance of the developed nanocomposite toward MG and Cd^2+^ was systematically investigated under different operational parameters, including pH, contact time, temperature, and initial pollutant concentration. The experimental results were analyzed using kinetic, isotherm, and thermodynamic models to elucidate the adsorption mechanisms. Collectively, the obtained results demonstrate that the **CuO@Pyridine PC** nanocomposite represents a stable, efficient, and sustainable adsorbent for the simultaneous removal of organic dyes and heavy metal ions from contaminated water systems.

## 2. Results

### 2.1. Synthetic Strategy and Reaction Pathway of Pyridine PC

**Scheme 1:**
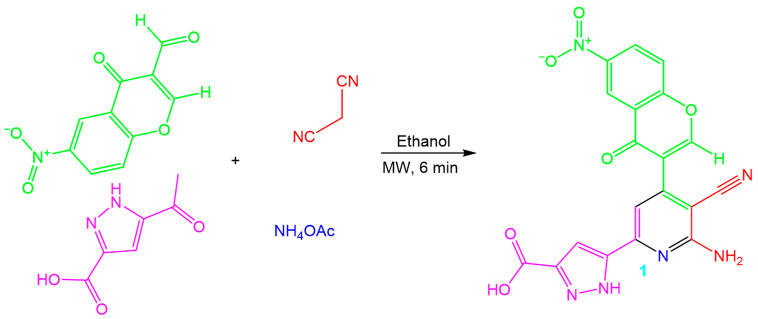
Synthesis of 2-aminonicotinonitrile **Pyridine PC** sorbent.

The **Pyridine PC** sorbent was constructed through a one-pot multicomponent reaction involving 6-nitro-4-oxo-4*H*-chromene-3-carbaldehyde, 5-acetyl-1*H*-pyrazole-3-carboxylic acid, malononitrile, and ammonium acetate, as illustrated in Scheme 1. The transformation proceeded efficiently under different activation modes, affording the target heterocyclic framework in high yield.

From a mechanistic perspective, the reaction follows a coherent and well-defined sequence. Initial in situ generation of the iminium/enamine intermediate occurs through the interaction of 5-acetyl-1*H*-pyrazole-3-carboxylic acid with ammonium acetate. Concurrently, a Knoevenagel condensation between the chromene aldehyde and malononitrile furnishes the highly electrophilic 2-((6-nitro-4-oxo-4*H*-chromen-3-yl)methylene)malononitrile intermediate. Subsequent nucleophilic addition of the enamine species, followed by intramolecular cyclization, isomerization, and final aromatization, leads to the formation of the fused pyridine–pyrazole–chromone heterosystem that defines the **Pyridine PC** framework.

This convergent multicomponent strategy enables efficient construction of the molecular architecture while minimizing synthetic steps, making it a practical route for preparing functional adsorbent materials.

### 2.2. Structural Characterization of the Prepared Materials

#### 2.2.1. Spectroscopic Confirmation of Pyridine PC

The successful construction of the **Pyridine PC** sorbent was unambiguously confirmed by comprehensive spectroscopic and analytical characterization. The ^1^H NMR spectrum recorded in DMSO-d_6_ (Figure S1) displays two markedly deshielded broad singlets at δ 12.18 and δ 11.36 ppm, attributable to the carboxylic OH and pyrazole NH protons, respectively. Aromatic proton resonances associated with the chromone and pyridine rings appear in the δ 8.61–7.32 ppm range, while the amino (NH_2_) protons are observed as a distinct singlet at δ 7.03 ppm. In addition, the characteristic pyrazole C–H proton resonates at δ 6.72 ppm, in full agreement with the proposed structure.

The ^13^C NMR spectrum (Figure S2) further substantiates the molecular framework, revealing two carbonyl carbon signals at δ 180.0 and δ 175.9 ppm. Signals corresponding to the sp^2^ carbons of the chromone and pyridine moieties are distributed throughout the δ 166–125 ppm region, while the nitrile carbon appears at δ 121.54 ppm, confirming the incorporation of the cyano functionality within the heterocyclic backbone.

Electron-impact mass spectrometry shows a molecular ion peak at *m*/*z* 418.08 (calcd. 418.07), consistent with the expected molecular weight of **Pyridine PC**. Elemental (CHN) analysis closely matches the theoretical values, validating the molecular formula C_19_H_10_N_6_O_6_. Collectively, the concordant NMR, mass spectrometric, and elemental analysis data provide conclusive evidence for the successful synthesis of the highly conjugated, heteroatom-rich **Pyridine PC** sorbent (Figure S3).

#### 2.2.2. X-Ray Diffraction (XRD) Analysis of the Prepared Materials

**Figure 1:**
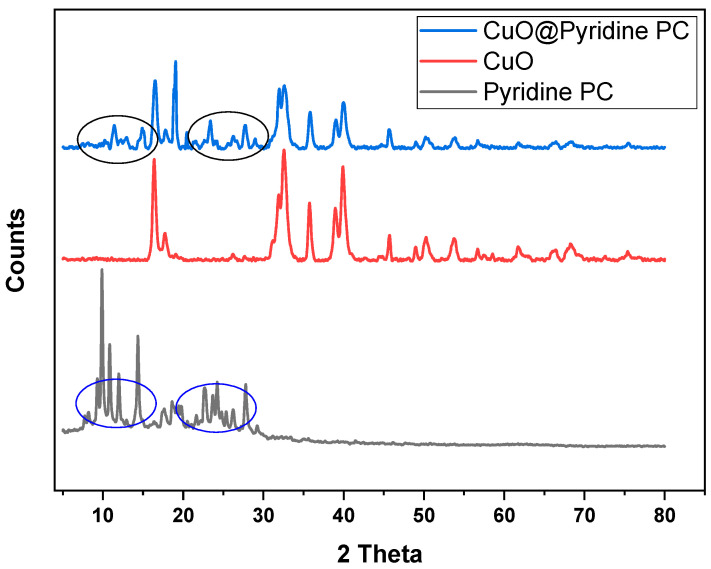
Comparative XRD patterns of **Pyridine PC**, CuO nanoparticles, and **CuO@Pyridine PC** composite. The highlighted regions correspond to diffraction features associated with Pyridine PC, which are significantly weakened or absent in the composite.

The comparative XRD patterns of **Pyridine PC**, CuO nanoparticles, and the **CuO@Pyridine PC** composite are presented in Figure 1.

The XRD pattern of **Pyridine PC** exhibits multiple sharp diffraction peaks, particularly in the low-angle region (2θ ≈ 10–25°), indicating that the material possesses a partially crystalline structure rather than being purely amorphous. This behavior suggests the presence of ordered domains within the heteroatom-rich organic framework.

In contrast, the synthesized CuO nanoparticles display several well-defined diffraction peaks at approximately 32.5°, 35.8°, 38.7°, 48.7°, 53.5°, 58.3°, and 61.6° (2θ), which can be indexed to the monoclinic tenorite phase of CuO (COD 1011194). The absence of additional peaks confirms the high purity of the synthesized CuO without detectable impurity phases such as Cu_2_O or metallic copper.

The average crystallite size of the CuO nanoparticles was estimated using the Scherrer equation based on the most intense peak at 2θ ≈ 35.8°, yielding an average crystallite size of approximately 17 nm, confirming their nanoscale nature.

For the **CuO@Pyridine PC** composite, the XRD pattern is predominantly governed by the characteristic diffraction peaks of CuO. However, significant changes are observed compared to the pristine **Pyridine PC** pattern. Specifically, the sharp peaks originally observed for **Pyridine PC** in the 2θ range of 10–25° become markedly weakened, broadened, or disappear in the composite.

These observations indicate that the original crystalline structure of **Pyridine PC** is not preserved after CuO incorporation. Instead, the structural features of **Pyridine PC** undergo modification, likely due to strong interfacial interactions between CuO nanoparticles and the organic matrix. Such interactions may include coordination between Cu species and nitrogen-containing functional groups, leading to disruption of the original ordering within the Pyridine PC framework.

Additionally, the slight reduction in CuO peak intensity and minor peak broadening in the composite can be attributed to the good dispersion of CuO nanoparticles on the **Pyridine PC** surface and possible size or strain effects induced by the interaction with the support.

### 2.3. Surface Area and Surface Charge Properties

#### 2.3.1. Brunauer–Emmett–Teller (BET) Surface Data

**Figure 2:**
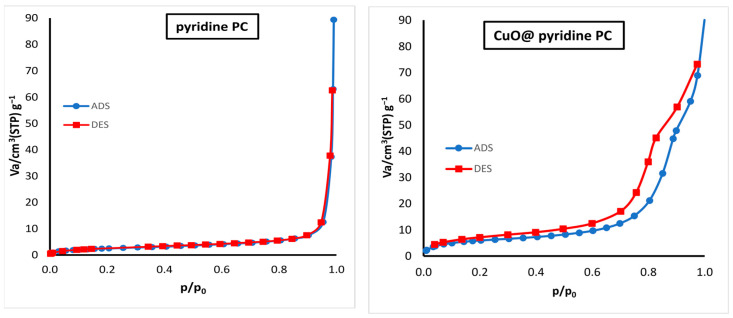
Nitrogen adsorption–desorption isotherms of **Pyridine PC** and **CuO@Pyridine PC** at 77 K.

Nitrogen adsorption–desorption measurements reveal a pronounced evolution in the textural properties of **Pyridine PC** following CuO incorporation, as evidenced by the N_2_ isotherms shown in Figure 2. The pristine **Pyridine PC** displays a moderate specific surface area of 9.1 m^2^ g^−1^, accompanied by a total pore volume of 0.128 cm^3^ g^−1^. These parameters indicate that adsorption on the unmodified sorbent is governed primarily by surface-accessible active sites together with the available pore structure.

Upon immobilization of CuO nanoparticles, the **CuO@Pyridine PC** composite exhibits a noticeable enhancement in surface characteristics. The specific surface area increases to approximately 19.8 m^2^ g^−1^, while the total pore volume expands to 0.138 cm^3^ g^−1^. Such changes reflect an improvement in porosity and surface accessibility, both of which are critical parameters governing adsorption capacity and kinetics.

In addition, the increased adsorption volume observed at higher relative pressures (p/p_0_ → 1) suggests the presence of interparticle voids and an expanded pore network after CuO loading. These structural modifications improve the accessibility of adsorption sites and are expected to contribute to the enhanced adsorption performance of the composite toward dye molecules and metal ions.

The pore size distribution of the prepared adsorbents was evaluated from nitrogen adsorption–desorption data and is presented in the Supplementary Information (Figure S4). The **Pyridine PC** exhibits dominant micropores with pore diameters mainly below 2–3 nm. Upon loading CuO nanoparticles, the distribution shifts toward larger pore sizes, with a noticeable contribution in the mesoporous range (8–15 nm). This behavior can be attributed to the deposition of CuO nanoparticles on the surface and within the pore structure, which partially blocks smaller micropores while generating interparticle voids and wider pores.

It is important to note that the pore structure is more accurately described by the pore size distribution rather than a single average pore diameter value. Accordingly, the material is characterized by a micropore-dominated structure in its pristine form, with the development of mesopores after CuO incorporation. The presence of mesopores (8–15 nm) is beneficial for adsorption processes, as it enhances mass transfer and facilitates the diffusion of relatively large molecules such as dye species and metal ions.

Despite the relatively moderate BET surface area of the prepared materials, the adsorption capacity toward Cd(II) remains remarkably high. This behavior indicates that adsorption is not governed solely by surface area but also by the availability and accessibility of heterogeneous active sites on the sorbent surface. The **Pyridine PC** framework contains several nitrogen- and oxygen-containing functional groups capable of interacting with Cd(II) ions through electrostatic attraction and surface coordination. Furthermore, the incorporation of CuO nanoparticles introduces additional active sites that enhance metal ion binding. Consequently, the synergistic contribution of these surface functionalities plays a dominant role in the adsorption process, enabling high adsorption capacities even at relatively moderate surface areas. Moreover, the maximum adsorption capacity was obtained at a relatively high initial Cd(II) concentration (200 mg L^−1^), which increases the driving force for adsorption and promotes efficient utilization of the available adsorption sites.

#### 2.3.2. Zeta Potential Analysis

The surface charge characteristics and colloidal stability of the **CuO@Pyridine PC** composite were evaluated through zeta potential measurements conducted in triplicate, with the mean value reported. As presented in Figure S5, the composite exhibits a zeta potential of −31.5 mV at near-neutral pH, indicative of a strongly negatively charged surface.

This pronounced negative surface charge is primarily attributed to Cu–O surface groups and hydroxyl functionalities associated with the CuO nanoparticles anchored onto the **Pyridine PC** matrix. Zeta potential values exceeding ±30 mV are generally recognized as indicative of good colloidal stability; therefore, the measured value confirms that the CuO nanoparticles are effectively stabilized on the sorbent surface, with minimal propensity for aggregation.

The high colloidal stability of **CuO@Pyridine PC** ensures uniform dispersion of the active CuO phase in aqueous media, thereby preserving continuous accessibility of adsorption sites. This stability plays a direct role in the enhanced adsorption performance observed for both malachite green and Cd(II) ions, as it facilitates sustained and homogeneous interactions between the sorbent surface and dissolved contaminants.

### 2.4. Morphological and Chemical Analysis Before and After Adsorption

#### 2.4.1. FT-IR Measurement Results

**Figure 3:**
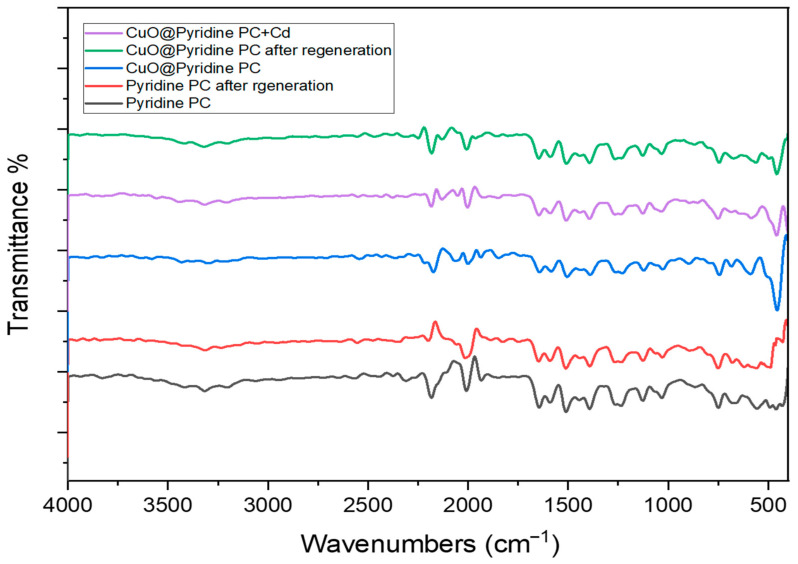
Comparative FT-IR spectra of **Pyridine-PC** and **CuO@Pyridine-PC** before and after five adsorption–desorption cycles, as well as after Cd^2+^ adsorption.

The FT-IR spectrum of **Pyridine PC** (Figure 3 and Figure S6) displays the characteristic vibrational features expected for its heteroatom-rich pyridine–pyrazole–chromone framework. A broad absorption envelope in the 3480–3250 cm^−1^ region is assigned to overlapping O–H and N–H stretching vibrations, consistent with the presence of hydroxyl- and amino-containing functionalities. The resolved bands at 3457 and 3336 cm^−1^ are mainly attributable to N–H stretching vibrations, whereas the band at 3089 cm^−1^ corresponds to aromatic C–H stretching, reflecting the conjugated aromatic skeleton of the sorbent. A sharp band at 2195 cm^−1^ is assigned to the nitrile (–C≡N) group, confirming retention of this key structural motif. In the fingerprint region, the bands at 1658 and 1620 cm^−1^ are assigned to C=O and C=N stretching vibrations, respectively, while the band at 1568 cm^−1^ is associated with aromatic C=C stretching. Additional absorptions at 1501 and 1226 cm^−1^ are attributable to N–O vibrations of the nitro group and C–O–C stretching of the pyran ring, respectively. Collectively, these bands confirm the successful formation of the **Pyridine PC** framework.

After CuO incorporation, the FT-IR spectrum of **CuO@Pyridine PC** shows a new band at approximately 470 cm^−1^, which is characteristic of Cu–O stretching and confirms the successful introduction of CuO into the organic matrix. In addition, moderate changes in the breadth and intensity of the O–H/N–H region suggest modification of the local hydrogen-bonding environment and interfacial interactions between CuO nanoparticles and the functional groups of **Pyridine PC**.

Following Cd(II) adsorption, noticeable shifts and intensity changes are observed for the O–H/N–H, C=O, and C=N bands. These spectral variations indicate the involvement of nitrogen- and oxygen-containing functional groups in Cd(II) binding. Although FT-IR alone does not define the adsorption pathway conclusively, the observed band changes, together with the EDX/elemental mapping results and the pH-dependent adsorption behavior, strongly support the contribution of surface coordination/complexation and electrostatic interactions during Cd(II) uptake. Therefore, the enhanced adsorption performance of **CuO@Pyridine PC** can be attributed to the synergistic presence of heteroatom-rich organic binding sites and additional CuO-derived surface functionalities.

#### 2.4.2. SEM–EDX and Elemental Mapping Analysis

**Figure 4:**
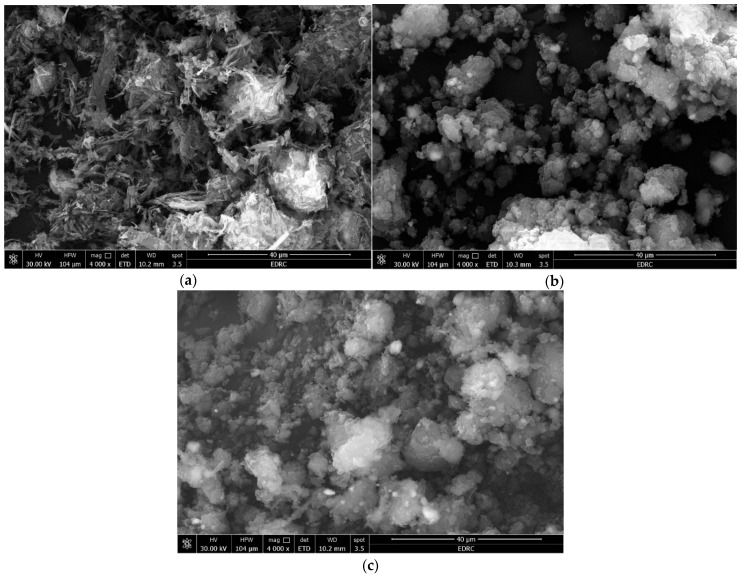
SEM images of pyridine-based sorbents at 4000× magnification: (**a**) **Pyridine PC**, (**b**) **CuO@Pyridine PC**, and (**c**) **CuO@Pyridine PC** after Cd^2+^ adsorption.

Scanning electron microscopy images of **Pyridine PC** recorded at 4000× magnification (Figure 4) reveal a distinctly rough and fibrous morphology composed of irregular flakes interconnected by open pores (Figure 4a). This hierarchical porous architecture provides extensive surface exposure and interconnected diffusion pathways, which are highly favorable for mass transfer and adsorption of metal ions. The uneven fibrous texture enables multiple interaction modes, including electrostatic attraction and surface complexation, rendering the pristine sorbent particularly effective for heavy-metal uptake.

Following CuO immobilization, pronounced morphological changes are observed (Figure 4b). The originally exposed fibrous framework becomes partially coated with nanoscale CuO particles, producing a more compact and granular surface appearance. The deposited nanoparticles markedly increase nanoscale roughness and introduce additional active sites, thereby enhancing the sorbent’s interaction potential with dissolved metal ions. Importantly, partial visibility of the underlying fibrous network indicates that the intrinsic porosity of **Pyridine PC** is largely preserved, ensuring continued accessibility of internal adsorption sites and efficient ion diffusion.

After Cd^2+^ adsorption, the surface of the **CuO@Pyridine PC** composite exhibits noticeable aggregation features (Figure 4c). Adhered nanoparticles and Cd^2+^-rich clusters are clearly visible, confirming strong interactions between the metal ions and the CuO-loaded sorbent. These morphological features indicate that adsorption occurs synergistically on both the CuO nanoparticle surfaces and the remaining exposed fibrous domains of the organic matrix. The combined effects of nanoparticle anchoring and metal-ion binding highlight the structural adaptability of the composite during adsorption and directly correlate with its enhanced removal efficiency.

**Figure 5:**
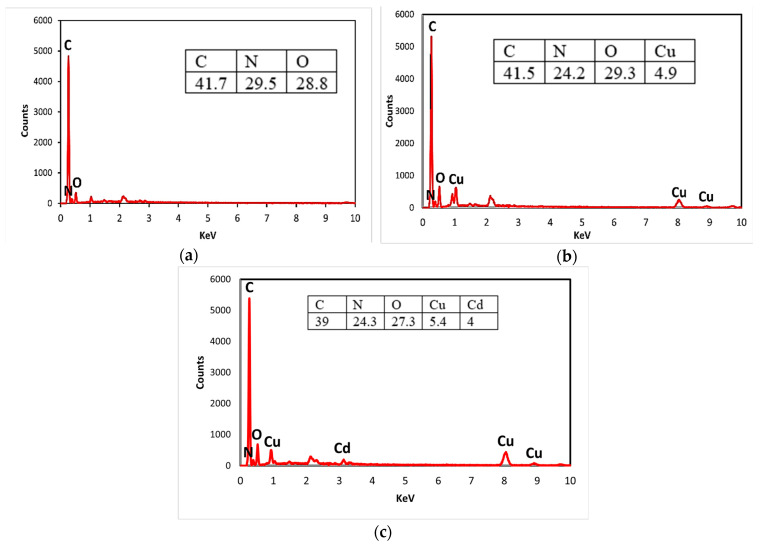
EDX spectra of (**a**) **Pyridine PC**, (**b**) **CuO@Pyridine PC**, and (**c**) **CuO@Pyridine PC** after Cd^2+^ adsorption.

Energy-dispersive X-ray spectroscopy further corroborates the SEM observations (Figure 5). The EDX spectrum of pristine **Pyridine PC** (Figure 5a) confirms its organic composition, dominated by carbon (41.7 wt%), nitrogen (29.5 wt%), and oxygen (28.8 wt%). The high nitrogen content reflects the abundance of pyridine- and pyrazole-type functionalities, which act as electron-donating centers and enhance affinity toward metal ions, while oxygen-containing groups contribute to surface polarity and adsorption activity. The absence of metallic signals verifies the purity of the organic sorbent prior to modification.

Upon CuO loading (Figure 5b), distinct copper signals appear, corresponding to a Cu content of 4.9 wt%, confirming successful immobilization of CuO nanoparticles. The relative decrease in nitrogen content suggests involvement of nitrogen-containing functional groups in anchoring CuO through surface interactions. Despite CuO incorporation, the dominance of C, N, and O signals indicates that the organic framework remains structurally intact, consistent with the SEM evidence of partial nanoparticle coverage while maintaining porous regions.

After Cd^2+^ adsorption (Figure 5c), characteristic cadmium peaks emerge, with a Cd content of 4.0 wt%, providing direct evidence of effective Cd^2+^ uptake. The concurrent reduction in C, N, and O percentages indicates occupation of available active sites by Cd^2+^ ions and their interaction with both CuO nanoparticles and heteroatom-rich functional groups of **Pyridine PC**. These findings confirm that cadmium adsorption proceeds through multiple mechanisms, including coordination and surface complexation, resulting in strong and stable metal binding.

**Figure 6:**
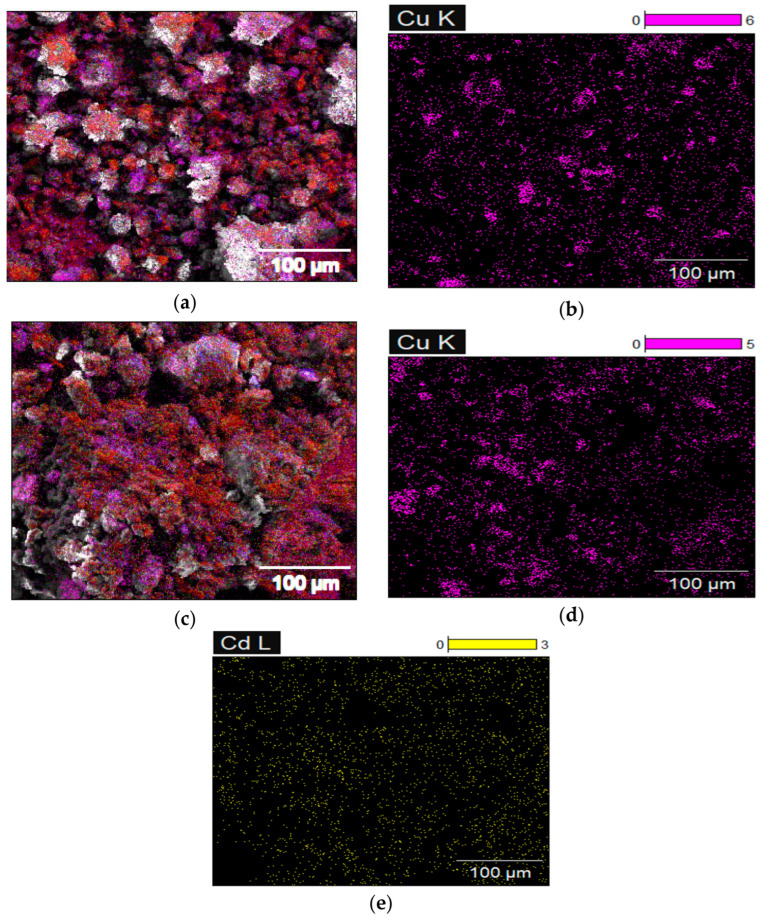
EDX elemental mapping of **CuO@Pyridine PC** composite: (**a**,**b**) Cu distribution before adsorption; (**c**–**e**) Cu and Cd(II) distribution after adsorption, demonstrating the successful adsorption of Cd(II) ions on the composite surface. Scale bar: 100 µm.

Figure 6 shows the elemental distribution of Cu and Cd^2+^ in the **CuO@Pyridine PC** composite was investigated using EDX mapping. The maps demonstrate a well-dispersed distribution of Cu across the composite surface before adsorption, while Cd^2+^ is clearly observed on the surface after adsorption, confirming successful binding of Cd(II) ions. These results support the effective interaction between the adsorbent and the target metal ions. Additional EDX mapping for C, N, and O elements is provided in the Supplementary Information (Figure S7), confirming the integrity of the pyridine framework and the presence of oxygen-containing functional groups.

### 2.5. Adsorption Performance

#### 2.5.1. Effect of pH on Adsorption Performance

**Figure 7:**
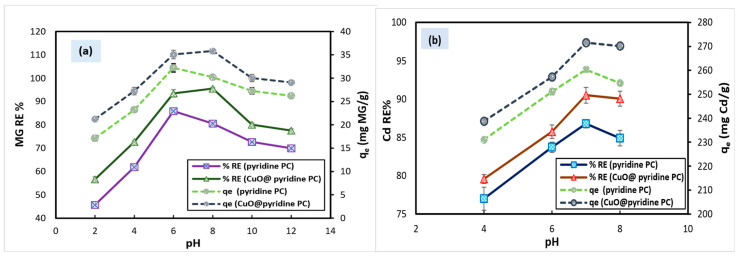
Effect of solution pH on adsorption capacity (q_e_, mg g^−1^) and removal efficiency (%) of (**a**) MG, and (**b**) Cd^2+^ by **Pyridine PC** and **CuO@Pyridine PC**. Error bars represent standard deviation (*n* = 3).

Solution pH plays a decisive role in controlling the adsorption behavior of malachite green (MG) and Cd^2+^ ions, as it directly governs sorbent surface charge and electrostatic interactions at the solid–liquid interface. The influence of pH on the adsorption performance of **Pyridine PC** and **CuO@Pyridine PC** was systematically investigated, and the results are summarized in Figure 7a,b.

For MG adsorption (Figure 7a), removal efficiency increases progressively with rising pH for both sorbents. **Pyridine PC** reaches its maximum removal efficiency (≈85.92%) at pH 6, whereas **CuO@Pyridine PC** exhibits superior performance, achieving ≈95.5% removal at pH 8. At acidic pH, adsorption is suppressed due to protonation of surface functional groups and strong competition between H^+^ ions and cationic dye molecules. As pH increases toward neutral and mildly alkaline conditions, surface deprotonation enhances electrostatic attraction between negatively charged sorbent sites and MG cations, leading to improved adsorption. The upward shift in optimum pH for the CuO-modified sorbent reflects altered surface charge characteristics and the contribution of additional active sites introduced by CuO nanoparticles.

A comparable pH-dependent trend is observed for Cd^2+^ adsorption (Figure 7b), albeit within a narrower pH window. **Pyridine PC** achieves a maximum cadmium removal efficiency of ≈86.77% at pH 7, while **CuO@Pyridine PC** reaches ≈ 90.55% after 1 h. At lower pH, Cd^2+^ uptake is limited by competition with protons for binding sites. Increasing pH promotes surface deprotonation and strengthens electrostatic attraction and coordination interactions with Cd^2+^ ions. Higher pH values were avoided to prevent cadmium hydroxide precipitation, which could confound adsorption measurements.

No visible precipitation was observed at pH 4–8. However, at pH 9, a white precipitate formed, and therefore this pH value was excluded from further experiments. Based on these observations, pH 7 was selected as the optimum condition for subsequent adsorption studies. Control experiments performed in the absence of sorbent confirmed negligible Cd(II) removal at pH 7 and 8, indicating that precipitation did not significantly contribute to metal removal under the studied conditions. The initial pH (pH_i_) was adjusted to the desired value prior to adsorption. The final pH (pH_f_) measured after equilibrium showed only minor variation (±0.2 units), remaining below the threshold for Cd(II) hydroxide precipitation.

These observations are fully consistent with pH_PZC_ values (Figure S8), which are approximately 5.0 for **Pyridine PC** and 5.5 for **CuO@Pyridine PC**. At pH values exceeding pH_PZC_, both sorbents acquire negatively charged surfaces that favor adsorption of cationic species such as MG and Cd^2+^. The consistently higher removal efficiencies of **CuO@Pyridine PC** highlight the synergistic role of surface charge development and CuO-derived active sites.

Although **CuO@Pyridine PC** shows maximum MG removal at pH 8, pH 6 was selected for subsequent dye adsorption experiments to maintain comparability with pristine **Pyridine PC** while preserving high composite performance.

#### 2.5.2. Effect of Contact Time and Kinetic Studies

**Figure 8:**
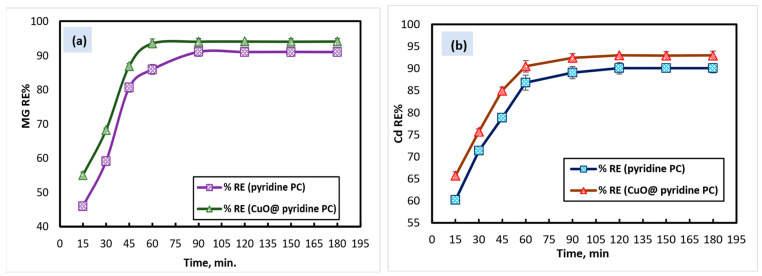
Effect of time on the removal efficiency and adsorption capacity of (**a**) MG and (**b**) Cd^2**+**^ ions by **Pyridine PC** and **CuO@Pyridine PC** at initial concentrations of 25 mg L^−1^ for MG (pH 6) and 200 mg L^−1^ for Cd^2+^ (pH 7), using an adsorbent dose of 0.05 g, a contact time of 1 h, and a temperature of ±25 °C. Error bars represent standard deviation (*n* = 3).

The influence of contact time on MG and Cd^2+^ adsorption was evaluated to elucidate adsorption kinetics and determine equilibrium time for **Pyridine PC** and **CuO@Pyridine PC** (Figure 8). For both pollutants, a rapid increase in adsorption capacity is observed during the initial stage, attributable to abundant available active sites and a steep concentration gradient between solution and sorbent. The intrinsic porosity of **Pyridine PC** promotes rapid mass transfer, while CuO incorporation further enhances surface heterogeneity and accessibility, accelerating initial uptake.

As contact time progresses, adsorption rates gradually decrease due to progressive occupation of active sites and reduced availability of energetically favorable locations. Repulsive interactions between adsorbed species and solutes remaining in solution may also contribute to this deceleration [24]. Equilibrium is effectively reached after ~90 min for both MG and Cd^2+^ on both sorbents. At this point, MG removal efficiencies reach ~91% and ~94% for **Pyridine PC** and **CuO@Pyridine PC**, respectively, while corresponding Cd^2+^ removals are ~90% and ~93%. Beyond 90 min, no appreciable increase in uptake is observed, indicating attainment of adsorption equilibrium.

**Figure 9:**
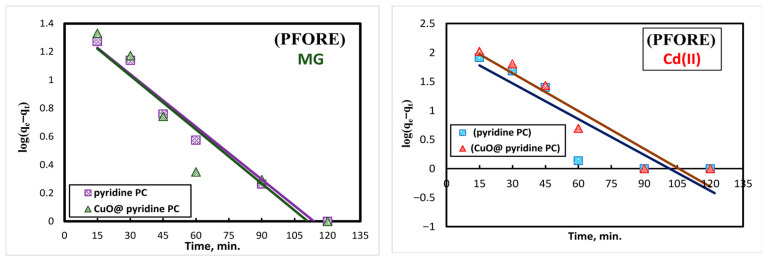
Pseudo-first-order kinetic model (PFORE) plots for MG and Cd^2+^ adsorption by **Pyridine PC** and **CuO@Pyridine PC** composite.

To elucidate the adsorption mechanism, kinetic data were analyzed using pseudo-first-order-equation (PFORE) and pseudo-second-order-equation (PSORE) models. PFORE plots (Figure 9, Table 1) show moderate correlation, particularly at early stages, and slightly higher rate constants (k_1_) for **CuO@Pyridine PC**, reflecting enhanced initial surface reactivity. However, calculated equilibrium capacities (q_e_,_cal_) are substantially lower than experimental values—especially for Cd^2+^ indicating that PFORE inadequately describes the full adsorption process.

**Figure 10:**
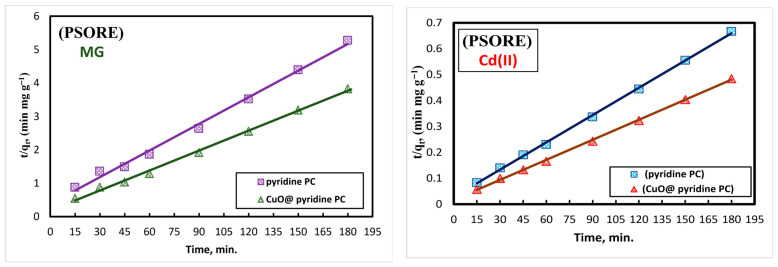
Kinetic model PSORE for MG and Cd(II) by **Pyridine PC** and **CuO@Pyridine PC** composite.

In contrast, PSORE modeling (Figure 10) provides excellent agreement with experimental data, with correlation coefficients exceeding 0.99 for all systems [25]. Calculated q_e_,_cal_ values closely match experimental capacities. For MG, q_e_,_cal_ increases from 37.6 to 50.3 mg g^−1^ upon CuO loading, while for Cd^2+^, a pronounced enhancement from 285.7 to 383.6 mg g^−1^ is observed. These results underscore the decisive role of CuO nanoparticles in supplying additional active sites and reinforcing surface interactions [26].

Overall, comparative kinetic analysis confirms that PSORE more accurately describes the adsorption behavior than PFORE, indicating that adsorption involves significant surface interactions such as coordination and surface complexation. However, when considered together with the Freundlich isotherm behavior and the relatively low enthalpy values, the adsorption process can be more appropriately described as a combined mechanism involving both chemisorption and physisorption contributions on heterogeneous surface sites. The synergistic contribution of nitrogen-containing functional groups and CuO-associated oxygen species accounts for the strong adsorption affinities observed.

#### 2.5.3. Effect of Initial Concentration and Adsorption Isotherm Studies

**Figure 11:**
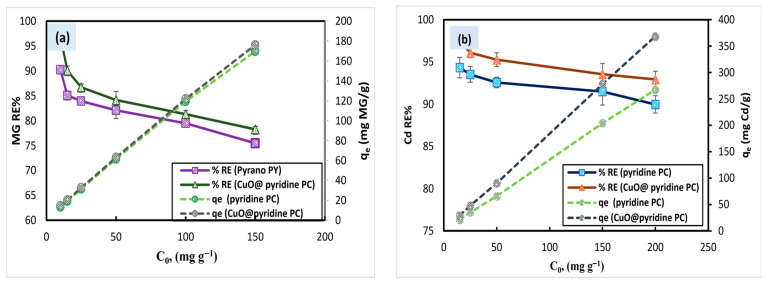
Effect of initial concentration on the adsorption performance of **Pyridine PC** and **CuO@Pyridine PC** toward (**a**) MG and (**b**) Cd^2+^ ions. Error bars represent standard deviation (*n* = 3).

The initial pollutant concentration is a key factor governing adsorption capacity, surface site utilization, and saturation behavior of adsorbent materials. Accordingly, the influence of initial malachite green (MG) concentration was evaluated over the range of 10–150 mg L^−1^ at room temperature using a fixed adsorbent dose of 0.05 g, as illustrated in Figure 11a.

At a low MG concentration of 10 mg L^−1^, high removal efficiencies of 90.2% and 96.6% were achieved for **Pyridine PC** and **CuO@Pyridine PC**, respectively, corresponding to equilibrium adsorption capacities of 13.53 and 14.5 mg g^−1^. This behavior reflects the abundance of available active sites relative to the number of dye molecules present in solutions. With increasing MG concentration beyond 15 mg L^−1^, a gradual decrease in removal efficiency was observed for both sorbents due to progressive saturation of accessible adsorption sites. In contrast, adsorption capacity increased continuously with increasing concentration, driven by the enhanced mass-transfer driving force. At an initial concentration of 150 mg L^−1^, maximum adsorption capacities of approximately 169.8 mg g^−1^ for **Pyridine PC** and 176.13 mg g^−1^ for **CuO@Pyridine PC** were obtained, clearly demonstrating the beneficial effect of CuO incorporation on dye uptake.

A similar concentration-dependent trend was observed for Cd^2+^ adsorption within the range of 15–200 mg L^−1^ (Figure 11b). At the lowest investigated concentration (15 mg L^−1^), removal efficiencies reached 94.33% for **Pyridine PC** and 97.47% for **CuO@Pyridine PC**, with corresponding q_e_ values of 21.25 and 29.24 mg g^−1^, respectively. Increasing the initial Cd^2+^ concentration led to a pronounced increase in adsorption capacity for both sorbents, reaching maximum values of approximately 267.47 mg g^−1^ for **Pyridine PC** and 368.32 mg g^−1^ for the CuO-loaded composite at 200 mg L^−1^. The markedly higher uptake of **CuO@Pyridine PC** confirms that CuO incorporation enhances both the density and effectiveness of active binding sites, particularly for heavy metal ion adsorption.

To elucidate adsorption mechanisms and surface characteristics, equilibrium data for MG and Cd(II) were analyzed using Langmuir, Freundlich, and Temkin isotherm models.

**Figure 12:**
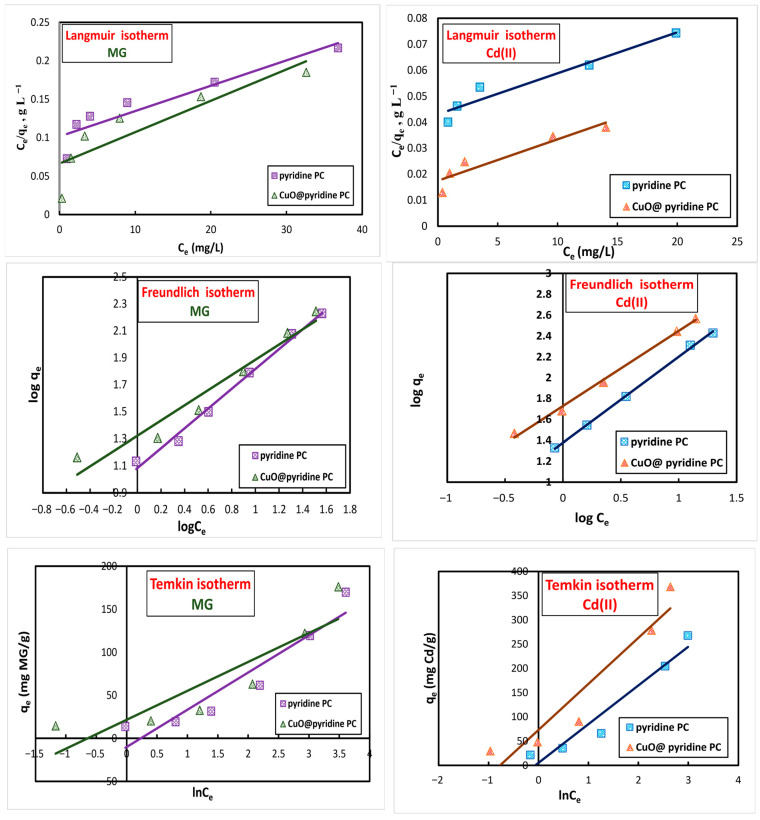
Adsorption isotherm models for MG dye and Cd(II) adsorption onto the prepared sorbents.

As shown in Figure 12 and summarized in Table 2, the Langmuir model exhibits moderate to good linearity for both adsorbates, with correlation coefficients ranging from 0.78 to 0.94. The calculated q_max_ values are consistently higher than the experimentally observed maximum capacities. For example, the Langmuir q_max_ for MG adsorption on **Pyridine PC** is 226 mg g^−1^ compared to an experimental value of 169.8 mg g^−1^, while a similar overestimation is observed for Cd(II). This deviation reflects the inherent limitations of the Langmuir assumptions, which do not account for surface heterogeneity or multilayer adsorption. Nevertheless, the obtained K_L_ values indicate appreciable affinity between the adsorbates and sorbent surfaces, particularly after CuO modification [27].

The Freundlich isotherm model was subsequently employed to assess adsorption intensity and surface heterogeneity. As depicted in Figure 12 and Table 2, the Freundlich model provides an excellent description of the experimental data, with correlation coefficients exceeding 0.94 for all systems and surpassing 0.99 in several cases. The *n* values greater than unity confirm favorable adsorption on heterogeneous surfaces. Notably, the Freundlich capacity constant (K_F_) increases substantially upon CuO loading, particularly for Cd(II), indicating enhanced surface affinity and stronger binding interactions. These findings suggest adsorption on energetically non-uniform sites with possible multilayer formation.

The Temkin isotherm model was also applied to evaluate adsorbate–adsorbent interaction energies and the influence of surface coverage on adsorption heat. The Temkin constants (K_T_ and B_T_), summarized in Figure 12 and Table 2, indicate moderate adsorption energies for both MG and Cd(II). Compared to pristine **Pyridine PC**, the **CuO@Pyridine PC** composite exhibits higher B_T_ values—particularly for Cd(II)—reflecting stronger interactions between metal ions and the modified surface. Although the Temkin model yields lower correlation coefficients than the Freundlich model, it supports the contribution of interaction-driven adsorption processes.

Overall, comparative isotherm analysis demonstrates that the Freundlich model provides the best description of the equilibrium adsorption behavior, indicating adsorption on heterogeneous surfaces with possible multilayer formation. This multilayer adsorption behavior suggests the contribution of physical interactions, including electrostatic attraction, π–π interactions, and van der Waals forces. Therefore, the overall adsorption mechanism reflects a synergistic combination of chemisorption and physisorption processes, consistent with both the kinetic and isotherm analyses. The Langmuir model was applied primarily to estimate the theoretical monolayer capacity (q_max_); however, its relatively lower correlation coefficients suggest that ideal monolayer adsorption is not the dominant mechanism. The Temkin model further supports the contribution of adsorbate–adsorbent interactions to the overall adsorption process [28].

The adsorption data for MG and Cd^2+^ on **Pyridine PC** and **CuO@Pyridine PC** were also analyzed using nonlinear Langmuir fitting to reduce linearization errors and obtain more accurate adsorption parameters. The higher q_max_ values obtained from nonlinear fitting can be attributed to the use of the original isotherm equation without data transformation, thereby minimizing distortion of the error structure. In contrast, linearized models may introduce deviations in adsorption parameters due to the mathematical transformation of the data.

However, it should be noted that the significantly higher q_max_ values obtained from nonlinear fitting may also result from the absence of a clear saturation plateau within the studied concentration range. Under such conditions, the Langmuir model extrapolates beyond the available experimental data, leading to an overestimation of the maximum adsorption capacity.

Therefore, a higher q_max_ value alone does not necessarily indicate a better representation of the adsorption process; rather, the model suitability is evaluated based on the overall fitting quality and its agreement with the experimental data.

**CuO@Pyridine PC** exhibited superior adsorption capacity compared to unmodified **Pyridine PC**, highlighting the beneficial effect of CuO loading. The nonlinear adsorption curves and detailed parameters are presented in Table 3 and Figure S9. Overall, the nonlinear fitting confirms the trends observed in the linear analysis while offering improved precision in q_max_ and K_L_ estimation.

To further improve the reliability of the isotherm analysis, the Freundlich model was also fitted using the nonlinear regression approach (Figure S10). The obtained parameters are presented in Table 3. In general, the nonlinear fitting provided correlation coefficients comparable to or slightly higher than those obtained from the linearized form, indicating a good agreement between the experimental data and the Freundlich model. For MG dye adsorption, the nonlinear Freundlich constants (K_F_) were 10.8 and 15.67 mg g^−1^ for **Pyridine PC** and **CuO@Pyridine PC**, respectively, with high correlation coefficients (R^2^ = 0.993–0.9989). Similarly, for Cd(II) adsorption, K_F_ values of 25.9 and 35.67 mg g^−1^ were obtained for **Pyridine PC** and **CuO@Pyridine PC**, respectively, with excellent fitting (R^2^ = 0.993–0.9985). Overall, the nonlinear regression results confirm the suitability of the Freundlich model for describing the heterogeneous adsorption behavior of the studied adsorbents.

#### 2.5.4. Effect of Temperature and Thermodynamic Parameters

The effect of temperature on the adsorption behavior of malachite green (MG) dye and Cd^2+^ ions onto **Pyridine PC** and **CuO@Pyridine PC** was systematically examined to assess the thermal sensitivity and energetic characteristics of the adsorption process. Batch adsorption experiments were carried out over a temperature range of 298–328 K under the previously optimized conditions (pH 6 for MG and pH 7 for Cd^2+^, contact time of 90 min, and adsorbent dosage of 0.05 g). Based on the preliminary optimization experiments,

The initial concentrations were fixed at 10 mg L^−1^ for MG and 15 mg L^−1^ for Cd^2+^ to ensure reliable comparison across different temperatures.

**Figure 13:**
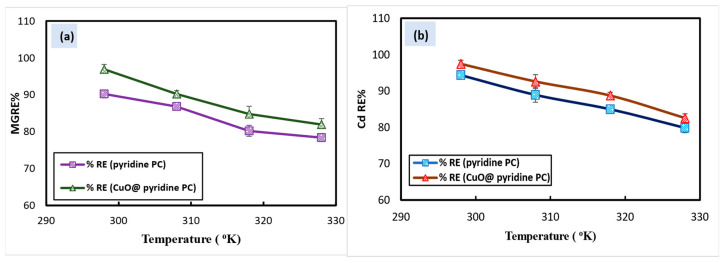
Effect of temperature on the adsorption efficiency of (**a**) MG and (**b**) Cd^2+^ by **Pyridine PC** and **CuO@Pyridine PC** composite. Error bars represent standard deviation (*n* = 3).

As shown in Figure 13, an increase in temperature led to a gradual decrease in the removal efficiency of both MG and Cd^2+^ ions for the two investigated sorbents. For MG adsorption, the removal efficiency of **Pyridine PC** decreased from 90.2% at 298 K to 78.4% at 328 K, while **CuO@Pyridine PC** exhibited higher efficiencies, declining from 96.9% to 81.8% over the same temperature range. A similar behavior was observed for Cd^2+^ adsorption, where **Pyridine PC** showed a decrease from 94.4% to 79.78%, whereas the composite sorbent demonstrated a comparatively smaller reduction from 97.47% to 82.5%.

The decrease in adsorption efficiency with increasing temperature suggests that the adsorption of both MG and Cd^2+^ ions is thermally unfavorable at elevated temperatures, which is consistent with an exothermic adsorption process. Higher temperatures may reduce adsorption capacity by increasing the kinetic energy of the adsorbate species, thereby weakening the interactions between the adsorbate and the active sites on the sorbent surface. Notably, **CuO@Pyridine PC** exhibits improved resistance to temperature-induced performance loss compared to the pristine sorbent, which may be attributed to the presence of CuO nanoparticles that modify surface properties and contribute to enhanced adsorption stability [29,30].

The thermodynamic parameters for MG adsorption onto **Pyridine PC** and **CuO@Pyridine PC** are summarized in Table 4 and Figure S11. The negative ΔG° values over the temperature range of 298–328 K confirm the spontaneous nature of the adsorption process for both sorbents. Moreover, the more negative ΔG° values observed for **CuO@Pyridine PC** indicate that the incorporation of CuO enhances the affinity of the sorbent toward MG molecules.

Similarly, the thermodynamic parameters for Cd(II) adsorption onto **Pyridine PC** and **CuO@Pyridine PC** are presented in Table 4 and Figure S11. The negative ΔG° values confirm the spontaneous adsorption of Cd(II) ions, while the more negative values obtained for the CuO-modified sorbent further indicate improved thermodynamic favorability after CuO incorporation. The negative ΔH° values suggest that the adsorption process is exothermic in nature.

For both adsorption systems, the negative ΔS° values indicate a decrease in randomness at the solid–solution interface during adsorption, suggesting a more ordered arrangement of the adsorbate molecules on the sorbent surface. This behavior may be attributed to the restriction of mobility of the adsorbate species and the formation of structured interactions with the active sites of the sorbent.

According to the thermodynamic relation (ΔG° = ΔH° − TΔS°), the negative entropy change opposes the adsorption process; however, the overall negative ΔG° values confirm that the process remains spontaneous under the studied conditions.

### 2.6. Desorption and Regeneration Results

The regeneration capability of an adsorbent is a decisive criterion for its practical application in wastewater treatment. Accordingly, the reusability of **Pyridine PC** and **CuO@Pyridine PC** was evaluated through successive adsorption–desorption cycles to assess structural stability and performance retention.

Desorption experiments were performed using eluents selected according to the nature of the adsorbed species. MG desorption was achieved using 0.2 M NaOH, which disrupts electrostatic and hydrogen-bonding interactions, whereas Cd(II) desorption was carried out using 0.1 M HCl, where protonation of surface functional groups weakens metal–surface coordination. solution under agitation for 60 min. The solid was separated by centrifugation, washed repeatedly with deionized water until neutral pH, and dried at 60 °C before reuse. After each cycle, eluates were neutralized and handled safely prior to disposal.

As summarized in Table 5, both adsorbents exhibit high desorption efficiencies for MG and Cd(II) over five consecutive cycles. Although a gradual decrease in desorption efficiency is observed with increasing cycle number, the values remain relatively high throughout. Notably, **CuO@Pyridine PC** maintains desorption efficiencies close to or exceeding 90%, while **Pyridine PC** retains efficiencies above 80% after five cycles. These results indicate good structural stability and predominantly reversible adsorption, with limited irreversible occupation of active sites.

Across all cycles, the CuO-loaded composite consistently outperforms the pristine sorbent, suggesting that CuO incorporation enhances surface robustness and facilitates reversible adsorption. Overall, these findings confirm that both materials possess excellent reusability and durability, with **CuO@Pyridine PC** emerging as a particularly promising and cost-effective adsorbent for repeated wastewater treatment applications.

To further evaluate the structural stability of the sorbents after regeneration, comparative FT-IR spectra of both **Pyridine PC** and **CuO@Pyridine PC** before and after the fifth adsorption–desorption cycle were recorded (Figure 3). The spectra show that the main characteristic functional groups are largely preserved after regeneration, with no significant shift in peak positions and only slight variations in band intensity. These minor changes may be attributed to adsorption–desorption interactions during repeated cycles. Importantly, the characteristic Cu–O vibration band remains clearly observable in the regenerated composite, confirming the continued presence of CuO within the structure.

Overall, the close similarity between the spectra before and after regeneration demonstrates that the essential functional groups of the sorbents are retained, indicating good structural stability during repeated regeneration cycles.

### 2.7. Application on Real Sample

The practical applicability of the synthesized **CuO@pyridine**-based nanocomposite was further evaluated using a real industrial wastewater sample collected from the effluent stream of a textile factory located in the 10th of Ramadan industrial zone, Cairo, Egypt. The sample was treated as received, without chemical pretreatment or pH adjustment, to simulate realistic operational conditions.

**Figure 14:**
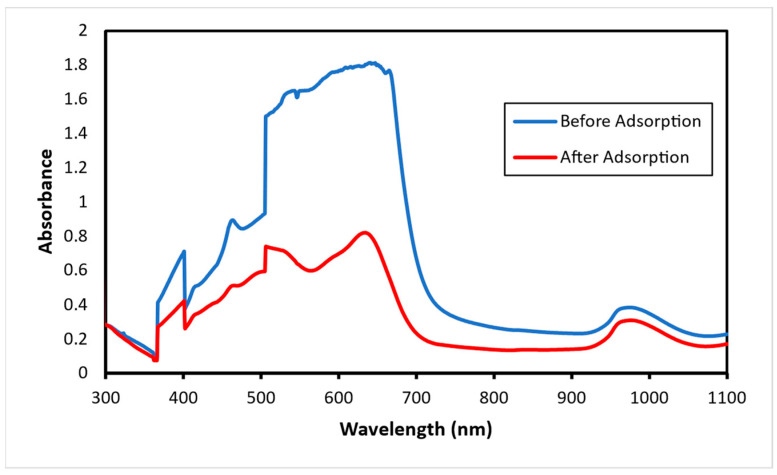
UV–Vis spectra of real textile wastewater before and after treatment with **CuO@Pyridine PC** nanocomposite.

The UV–Vis spectrum of the untreated wastewater (Figure 14) exhibits a broad absorption band across the visible region (400–750 nm), indicative of a complex mixture of dye species rather than a single chromophore. Such spectral characteristics are typical of textile effluents containing multiple cationic and basic dyes with overlapping absorption features.

Following treatment of 100 mL wastewater with 0.5 g of **CuO@Pyridine PC**, a substantial reduction in absorbance intensity is observed throughout the visible region. At the dominant wavelength (~620 nm), absorbance decreases from approximately 1.80 to 0.78, corresponding to an overall dye removal efficiency of about 57%. Given the complex composition of real textile effluents, removal efficiency was assessed based on the relative attenuation of the dominant absorption band.

The pronounced suppression of absorbance across the entire visible spectrum confirms the simultaneous removal of multiple dye species rather than selective adsorption of a single compound. These results demonstrate the effectiveness and robustness of the **CuO@Pyridine PC** nanocomposite under realistic wastewater conditions, highlighting its strong potential for real-world applications involving complex pollutant matrices.

The sharp increase in absorbance between ~380–400 nm is due to instrumental effects (e.g., lamp switching or detector interval adjustment) and does not reflect a physical transition of the dyes. This artifact does not affect the overall adsorption trend.

### 2.8. Comparative Evaluation of Cadmium and Malachite Green Adsorption by Pyridine PC and CuO@Pyridine PC Composite Relative to Literature Reports

To benchmark the adsorption performance of **Pyridine PC** and **CuO@Pyridine PC**, their maximum uptake capacities toward Cd(II) ions and malachite green (MG) dye were compared with representative adsorbents reported recently in the literature. The maximum adsorption capacity (q_max_) is widely used as a primary metric for evaluating adsorbent efficiency under optimized batch conditions. Recent studies show that engineered materials—such as modified carbons, functionalized nanomaterials, and designed hybrid composites—can exhibit high adsorption capacities; however, performance varies markedly with surface chemistry, pore architecture, and the abundance/accessibility of functional binding sites. For MG, exceptionally high capacities (often >700 mg g^−1^) have been reported for advanced adsorbents (e.g., functionalized porous polymers), emphasizing the role of large accessible surface areas and tailored π–π interactions in dye capture. In contrast, many conventional and low-cost sorbents used for Cd(II) typically exhibit capacities ranging from tens to low hundreds of mg g^−1^, with strong dependence on activation and functionalization strategies.

Table 6 compiles selected literature-reported adsorption capacities for MG and Cd(II) from recent peer-reviewed studies. This comparison contextualizes the present materials and highlights the competitive position of **CuO@Pyridine PC** within recent advances in adsorbent design.

This comparison indicates that, for MG, certain highly engineered polymeric and cyclodextrin-based matrices can achieve very high capacities, consistent with adsorption dominated by strong π-driven interactions and highly accessible adsorption domains. Nevertheless, **CuO@Pyridine PC** delivers a substantial capacity (~176 mg g^−1^), which is competitive with many conventional adsorbents and exceeds typical low-cost materials such as nanobentonite under comparable operating logic. For Cd(II), **Pyridine PC** and particularly **CuO@Pyridine PC** exhibit capacities in the high hundreds of mg g^−1^, surpassing many activated carbons and alkali-activated materials that often remain below ~100 mg g^−1^, and exceeding values reported for specialized inorganic adsorbents such as synthetic Na X zeolite (~185–268 mg g^−1^). The enhanced performance of **CuO@Pyridine PC** is attributed to synergistic coupling between the heterocyclic framework—rich in N/O coordination sites—and the CuO phase, which introduces additional binding domains and modifies surface charge, collectively enabling stronger and multi-mechanistic interactions with both MG and Cd(II). This benchmarking therefore supports the high practical relevance of the developed composite within the current landscape of water-pollutant adsorption research [39].

### 2.9. Experimentally Supported Adsorption Mechanism for Malachite Green and Cd(II) Uptake by Pyridine PC and CuO@Pyridine PC

**Scheme 2:**
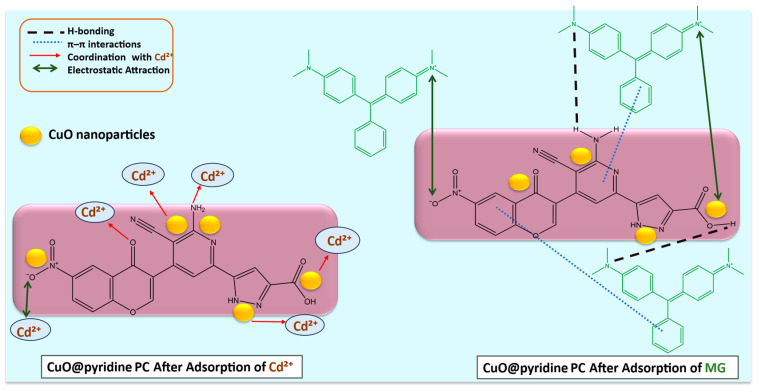
Schematic illustration of the experimentally supported adsorption pathways of MG dye and Cd(II) ions on **CuO@Pyridine PC**.

As illustrated in Scheme 2, the adsorption of malachite green (MG) and Cd(II) by **Pyridine PC** and **CuO@Pyridine PC** is governed by a cooperative mechanism arising from the heteroatom-rich organic framework and, in the composite, the additional CuO-derived surface sites. This interpretation is well supported by FT-IR, SEM/EDX, elemental mapping, BET analysis, and adsorption studies, including pH effect, kinetics, isotherms, and thermodynamics.

For **Pyridine PC**, the adsorption performance mainly originates from the abundance of nitrogen- and oxygen-containing functional groups within the heterocyclic framework, which provides effective binding sites. The pH-dependent behavior highlights the important role of surface charge, where increasing negative charge at pH > pH_PZC_ enhances electrostatic attraction toward cationic MG and Cd(II).

Cd(II) adsorption is primarily governed by coordination with N- and O-containing groups, as confirmed by FT-IR shifts in O–H, N–H, C=N, and C=O regions, along with EDX and mapping results indicating Cd presence on the surface. The good fit to the pseudo-second-order model further supports the dominance of strong surface interactions.

For MG, adsorption involves electrostatic attraction, hydrogen bonding, and π–π interactions between the aromatic dye and the conjugated framework. This is consistent with the Freundlich isotherm model, indicating adsorption on heterogeneous surface sites.

The enhanced performance of **CuO@Pyridine PC** compared with pristine Pyridine PC reflects a synergistic effect between the organic matrix and CuO nanoparticles. The incorporation of CuO increases surface area, pore volume, and surface heterogeneity, as supported by BET and SEM results. These changes improve accessibility and increase the density of active sites. CuO also introduces additional binding sites for Cd(II) and enhances electrostatic interactions and surface polarity for MG adsorption.

Overall, adsorption proceeds through a combined mechanism involving electrostatic attraction, coordination, hydrogen bonding, and π–π interactions. The coexistence of these pathways, together with the increased number of accessible active sites after CuO incorporation, accounts for the superior adsorption performance of the composite.

### 2.10. Economic Feasibility and Practical Implications

The economic and operational feasibility of **Pyridine PC** and **CuO@Pyridine PC** was systematically evaluated in the context of practical wastewater treatment implementation. Although activated carbon remains a widely adopted benchmark adsorbent owing to its high surface area and established industrial reliability, contemporary sustainability assessments indicate that adsorption efficiency alone does not determine practical competitiveness. Instead, production cost, regeneration requirements, energy demand, material durability, and lifecycle performance collectively define real-world economic viability. Commercial activated carbon typically exhibits a market price in the range of approximately 8–20 USD kg^−1^, depending on activation method, surface area, and material grade, which may influence its economic feasibility in large-scale or cost-sensitive treatment applications [40].

Conventional activated carbon production commonly involves high-temperature activation processes and, in many operational scenarios, energy-intensive thermal regeneration. These steps cumulatively increase the overall treatment cost across repeated adsorption–desorption cycles. In contrast, the materials developed in the present study were synthesized through a high-yield one-pot route under mild reaction conditions using readily accessible precursors. The absence of additional activation steps reduces processing intensity, energy consumption, and purification complexity, thereby improving the scalability potential of the synthesis strategy.

A defining feature of the developed composite is the transformation of discarded electrical copper wires into functional CuO nanoparticles. Rather than relying on virgin copper sources or commercially manufactured nanomaterials, post-consumer copper waste was directly converted into an active adsorption component and reintegrated into a value-generating environmental application. This strategy represents a waste valorization–driven resource recovery pathway that extends the functional lifespan of copper beyond its initial electrical application while reducing dependence on primary copper extraction and refining. Although the process does not constitute a fully closed metallurgical cycle, it reflects a structured material-flow intervention in which secondary resources are upgraded into higher-value functional components within an environmental remediation system.

Based on laboratory-scale raw material estimations, **Pyridine PC** can be produced at approximately 3–5 USD kg^−1^, whereas **CuO@Pyridine PC** is estimated at approximately 4–6 USD kg^−1^. When adsorption performance is incorporated into the economic assessment, the performance-adjusted cost becomes particularly competitive. **CuO@Pyridine PC** exhibits a maximum Cd(II) adsorption capacity of 368 mg g^−1^, which lies within the upper range of capacities reported for advanced carbon-based adsorbents depending on activation and surface modification strategies. The higher uptake capacity reduces the required adsorbent dosage per unit mass of contaminant removed, thereby lowering material consumption and operational cost per treated water volume.

Operational durability further strengthens economic feasibility. Both materials demonstrate rapid adsorption kinetics under ambient conditions and retain ≥90% regeneration efficiency across multiple adsorption–desorption cycles using dilute, low-cost eluents (HCl for Cd(II) and NaOH for MG), without requiring high-temperature thermal reactivation. When regeneration energy demand, service lifetime, and repeated-cycle performance are evaluated collectively within a lifecycle framework, the developed composite demonstrates strong economic competitiveness.

Overall, the combination of simplified synthesis, waste-derived resource recovery, enhanced adsorption capacity, and sustained reusability positions **CuO@Pyridine PC** as a technically robust and economically competitive adsorbent for the sustainable removal of MG and Cd(II) from contaminated water systems.

## 3. Discussion

The adsorption behavior of malachite green (MG) dye and Cd(II) ions onto **Pyridine PC** and **CuO@Pyridine PC** can be explained by the combined effects of surface chemistry, electronic structure, and textural properties of the developed materials. The adsorption results, including kinetics, thermodynamics, and spectroscopic analyses, indicate that the process is governed by cooperative physicochemical interactions rather than a single mechanism.

For **Pyridine PC**, adsorption mainly arises from the abundance of heteroatom-containing functional groups within the heterocyclic framework. Nitrogen-containing moieties such as pyridinic, pyrazole, amino, and cyano groups, together with oxygen-containing sites including carbonyl and phenolic functionalities, provide multiple electron-donating centers capable of interacting with cationic pollutants. In the case of Cd(II), these groups facilitate surface complexation through metal–ligand coordination, consistent with the spontaneous adsorption behavior and the good agreement with the pseudo-second-order kinetic model.

For MG adsorption, electrostatic attraction between the negatively charged surface and the cationic dye molecules plays a key role. In addition, the π-conjugated domains of the heterocyclic framework enable π–π interactions with the aromatic structure of MG, while hydrogen bonding involving surface –NH, –OH, and C=O groups further stabilizes the adsorption process. The coexistence of these interactions accounts for the high adsorption performance even with a moderate surface area.

Incorporation of CuO into the **Pyridine PC** matrix significantly enhances adsorption performance due to a synergistic organic–inorganic effect. The CuO phase introduces additional oxygen-rich binding sites and modifies the surface charge distribution, increasing the diversity and density of active adsorption centers. Consequently, Cd(II) adsorption benefits from simultaneous coordination with Cu–O sites and interaction with heteroatom groups of the organic framework.

Similarly, for MG removal, the presence of CuO improves surface polarity and charge heterogeneity, strengthening electrostatic attraction and hydrogen-bonding interactions with dye molecules. These effects result in a more efficient and multimodal adsorption pathway, consistent with the improved adsorption capacity of the composite.

The structural characteristics revealed by BET and SEM analyses further support these mechanisms. Dispersion of CuO within the **Pyridine PC** matrix increases surface roughness and accessibility of active sites, facilitating mass transfer and improving pollutant–surface interactions.

The practical applicability of the composite is demonstrated by its performance in treating real textile wastewater, where significant attenuation of absorbance across the visible spectrum confirms the effective removal of multiple dye species from complex effluents.

Comparative analysis with previously reported adsorbents indicates that the developed materials exhibit competitive adsorption performance. In particular, the Cd(II) adsorption capacity of **CuO@Pyridine PC** surpasses many conventional activated carbons and several reported inorganic adsorbents, highlighting the effectiveness of combining heterocyclic frameworks with metal oxide phases.

The adsorption behavior of malachite green (MG) dye and Cd(II) ions onto **Pyridine PC** and **CuO@Pyridine PC** can be interpreted entirely on the basis of the experimental dataset. The combined evidence from FT-IR, SEM/EDX, elemental mapping, BET analysis, pH-dependent adsorption, kinetic modeling, isotherm fitting, and thermodynamic evaluation demonstrates that adsorption proceeds through a cooperative mechanism involving electrostatic attraction, surface complexation/coordination, hydrogen bonding, and π–π interactions. The enhanced performance of **CuO@Pyridine PC** is attributed to the synergistic integration of the heteroatom-rich **Pyridine PC** framework with CuO-derived active sites, which increases surface heterogeneity, improves accessibility of adsorption centers, and strengthens pollutant–surface interactions. These findings are fully consistent with the superior adsorption capacity and practical wastewater-treatment performance of the composite.

Overall, adsorption of MG and Cd(II) on **CuO@Pyridine PC** occurs through a cooperative mechanism involving electrostatic attraction, π–π interactions, hydrogen bonding, and surface complexation. The synergy between the heterocyclic organic framework and the CuO phase accounts for the enhanced adsorption performance and demonstrates the potential of the composite for wastewater treatment applications.

## 4. Materials and Methods

### 4.1. Materials

All reagents and solvents were obtained from commercial suppliers and used as received. The purity of the synthesized adsorbent (**Pyridine PC**) and reaction progress were monitored by thin-layer chromatography (TLC) using Merck silica gel 60 F254 aluminum plates and visualization under UV light (254 nm). Analytical-grade malachite green (MG) dye and cadmium nitrate tetrahydrate (Cd(NO_3_)_2_·4H_2_O) were employed as representative cationic organic and inorganic contaminants, respectively. Nitric acid was used during preparation and treatment steps, and post-consumer copper wires served as the copper source. Solution pH was adjusted using hydrochloric acid (HCl) and sodium hydroxide (NaOH). Unless otherwise stated, chemicals were of analytical grade and purchased from Sigma-Aldrich. (St. Louis, MO, USA).

### 4.2. Preparation of Pyridine PC

The target sorbent, 5-(6-amino-5-cyano-4-(6-nitro-4-oxo-4*H*-chromen-3-yl)pyridin-2-yl)-1*H*-pyrazole-3-carboxylic acid (abbreviated herein as **Pyridine PC**), was synthesized as follows:

*Method A (Conventional Heating):* 6-nitro-4-oxo-4*H*-chromene-3-carbaldehyde (0.01 mol, 2.19 g), 5-acetyl-1*H*-pyrazole-3-carboxylic acid (0.01 mol, 1.54 g), ammonium acetate (0.08 mol, 6.2 g), and malononitrile (0.015 mol, 0.99 g) were dissolved in ethanol (50 mL) and refluxed for 5 h. After cooling to room temperature, the precipitate was filtered and purified by recrystallization from ethanol.

*Method B (Microwave-Assisted Synthesis):* 5-acetyl-1*H*-pyrazole-3-carboxylic acid (0.01 mol, 1.54 g), 6-nitro-4-oxo-4*H*-chromene-3-carbaldehyde (0.01 mol, 2.19 g), malononitrile (0.01 mol, 0.66 g), and ammonium acetate (0.04 mol, 3.07 g) were subjected to microwave irradiation (270 W) for 6 min. The mixture was cooled, and the solid product was collected, washed with ethanol, dried, and recrystallized from ethanol.

*Method C (Ultrasonic Irradiation):* 6-nitro-4-oxo-4*H*-chromene-3-carbaldehyde (0.01 mol, 2.19 g), 5-acetyl-1*H*-pyrazole-3-carboxylic acid (0.01 mol, 1.54 g), ammonium acetate (0.04 mol, 3.07 g), and malononitrile (0.01 mol, 0.66 g) were dissolved in ethanol (10 mL) and ultrasonicated at room temperature for 20 min with TLC monitoring. The resulting solid was filtered, thoroughly washed with ethanol, and dried to yield a pale off-white powder.

### 4.3. Synthesis of Copper Oxide (CuO) Nanoparticles from Scrap Copper Wire

CuO nanoparticles were prepared from discarded electrical copper wires via wet-chemical precipitation followed by thermal conversion. The wires were separated from insulation, washed with distilled water, cleaned with acetone, and cut into small pieces. Copper pieces were dissolved in 2 M nitric acid under stirring to obtain a clear blue copper nitrate solution. After filtration, the solution was titrated dropwise with 1.0 M NaOH until a light blue precipitate formed at ~pH 8. The precipitate was repeatedly washed with deionized water to neutrality, dried at 80 °C, and calcined at 300 °C for 3 h to obtain CuO nanoparticles, which were ground and stored for subsequent use.

### 4.4. Loading of CuO Nanoparticles onto Pyridine PC

A CuO-loaded composite containing 20 wt% CuO was produced by incorporating the synthesized CuO nanoparticles into **Pyridine PC**. **Pyridine PC** was dispersed in deionized water under continuous stirring to ensure hydration and activation of functional groups. The required CuO mass was gradually added, followed by sonication for 30 min to break agglomerates and promote uniform dispersion of the nanoparticles.

Subsequently, the mixture was magnetically stirred for 4 h at room temperature to facilitate effective interaction and anchoring of CuO nanoparticles onto the sorbent surface. The resulting composite was separated by centrifugation, washed several times with deionized water to remove loosely bound particles, and dried at 60 °C until constant weight.

### 4.5. Adsorbate Preparation

A malachite green stock solution (1000 mg L^−1^) was prepared by dissolving 1.0 g malachite green oxalate in 1 L distilled water and stored in an amber bottle to minimize photodegradation. A cadmium stock solution (1000 mg L^−1^) was prepared by dissolving 2.74 g Cd(NO_3_)_2_·4H_2_O in distilled water. Where required, solution pH was adjusted using 0.1 M NaOH or 0.1 M HCl and measured using a calibrated pH meter. (Mettler Toledo, Greifensee, Switzerland).

### 4.6. Instrumentation

^1^H and ^13^C NMR spectra were recorded on a JEOL ECA-500 II instrument (JEOL Ltd., Tokyo, Japan) (500 and 125 MHz, respectively) using DMSO-d_6_. Mass spectra were obtained using a SHIMADZU GC/MS instrument (Shimadzu Corporation, Kyoto, Japan). Elemental (CHN) analysis was conducted using a Vario EL III Elementar Analyzer. (Elementar Analysensysteme GmbH, Langenselbold, Germany) Brunauer–Emmett–Teller (BET) surface area analysis was carried out using a BELSORP MINI X analyzer (MicrotracBEL Corp., Osaka, Japan) at Mansoura University. Prior to measurement, all samples were degassed at 110 °C for 6 h under vacuum to remove moisture and physically adsorbed gases. Nitrogen adsorption–desorption isotherms were recorded at 77 K. Surface morphology was examined using a JSM-7500FA (JEOL Ltd., Tokyo, Japan) high-resolution cold field emission SEM, and elemental composition was determined by EDX. FT-IR spectra were recorded using a Nicolet iS10 FT-IR spectrophotometer (Thermo Fisher Scientific, Waltham, MA, USA) spectrophotometer. UV–Vis measurements were performed using a Shimadzu UV-2450 spectrophotometer. The maximum absorption wavelength (λmax) of Malachite Green (MG) was determined at 617 nm and used for all measurements. The concentration of Cd(II) was determined using an atomic absorption spectrophotometer (AAS, SENSAA Dual, GBC Scientific Equipment Pty Ltd., Melbourne, Australia) using the calibration method. In addition, the material and volume of the tubes used in the adsorption experiments (glass tubes, 50 mL) have been clearly indicated in the revised manuscript.

### 4.7. Batch Adsorption Experiments

Batch adsorption experiments were conducted to evaluate the adsorption behavior of **Pyridine PC** and **CuO@Pyridine PC** as a function of pH, initial concentration, contact time, and temperature. Experiments were performed in tightly sealed tubes using a temperature-controlled shaker at 150 rpm. Temperatures were maintained at 25, 35, 45, or 55 °C as required.

#### 4.7.1. Effect of pH

Solution pH was adjusted using 0.1 M HCl or 0.1 M NaOH. For MG, pH values ranged from 2 to 12; for Cd^2+^, pH was set to 4, 6, 7, and 8. In each run, 0.05 g sorbent was added to 75 mL solution containing 25 mg L^−1^ MG or 200 mg L^−1^ Cd^2+^. Suspensions were agitated at 150 rpm and 25 °C for 120 min (equilibrium contact time). Samples were filtered and residual concentrations were determined to assess adsorption performance.

#### 4.7.2. Effect of Contact Time

Kinetic experiments were performed using initial concentrations of 25 mg L^−1^ (MG) and 200 mg L^−1^ (Cd^2+^). In each run, 0.05 g sorbent was added to 75 mL solution at the respective optimum pH. Tubes were shaken at 150 rpm and 25 °C, and aliquots were collected at 15, 30, 45, 60, 90, 120, 150, and 180 min. Samples were filtered and analyzed to determine uptake rate and equilibrium time.

#### 4.7.3. Effect of Initial Concentrations

MG and Cd^2+^ solutions (15–200 mg L^−1^) were prepared from stock solutions. A fixed sorbent dose (0.05 g) was added to 75 mL of each solution, followed by shaking at 150 rpm and 25 °C for 90 min. After filtration, residual concentrations were measured to evaluate the effect of initial concentration.

#### 4.7.4. Effect of Temperature

Temperature-dependent experiments were carried out at 25, 35, 45, and 55 °C using MG (10 mg L^−1^) and Cd^2+^ (25 mg L^−1^) solutions. Experiments were conducted at pH 6 (MG) and pH 7 (Cd^2+^), with 0.05 g sorbent per 75 mL solution, at 150 rpm in a temperature-controlled incubator shaker.

The adsorption capacity at equilibrium (q_e_, mg g^−1^) and removal efficiency (%R) were calculated using Equations (1) and (2), respectively:

(1) $$(q_{e})\;=\frac{(Co-Ce)\times V}{m}$$

(2) $$R\%=(\frac{Co-Ce}{Co})\times 100$$

where m is the sorbent mass (g), V is the solution volume (L), and C_0_ and C_e_ are the initial and equilibrium concentrations (mg L^−1^), respectively. All experiments were conducted in triplicate (*n* = 3). Results are presented as mean ± standard deviation. Error bars in the figures represent the standard deviation of three independent measurements [41].

Control experiments were conducted under identical experimental conditions in the absence of sorbent to exclude possible Cd(II) precipitation and dye photodegradation. Negligible changes in concentration were observed, confirming that removal occurred predominantly via adsorption.

#### 4.7.5. Adsorption Isotherm and Kinetic Modeling

The adsorption equilibrium data were analyzed using the Langmuir, Freundlich, and Temkin isotherm models to describe the interaction between the adsorbate and the adsorbent surface. The adsorption kinetic behavior was evaluated using the pseudo-first-order and pseudo-second-order kinetic models. Both linear and nonlinear forms of these models were applied to analyze the experimental data. However, the model parameters were estimated by fitting the experimental data to the original nonlinear forms of the equations using nonlinear regression analysis.

The nonlinear form of the Langmuir isotherm model is expressed as:

(3) $$q_{e}=\frac{q_{max}K_{L}C_{e}}{1+K_{L}C_{e}}$$

where $q_{e}$ (mg g^−1^) is the adsorption capacity at equilibrium, $q_{max}$ (mg g^−1^) is the maximum monolayer adsorption capacity, $K_{L}$ (L mg^−1^) is the Langmuir constant related to the affinity of binding sites, and $C_{e}$ (mg L^−1^) is the equilibrium concentration of the adsorbate.

The Freundlich model is represented as:

(4) $$q_{e}=K_{F}C_{e}^{1/n}$$

where $K_{F}$ and n are the Freundlich constants related to adsorption capacity and adsorption intensity, respectively.

The Temkin isotherm model can be expressed as:

(5) $$q_{e}=\frac{\mathrm{RT}}{B_{T}}\ln K_{T}C_{e}$$

where R is the universal gas constant (8.314 J mol^−1^ K^−1^), T is the absolute temperature (K), $K_{T}$ is the Temkin equilibrium binding constant, and $B_{T}$ is related to the heat of adsorption.

The pseudo-first-order kinetic model is expressed as:

(6) $$q_{t}=q_{e}(1-e^{-k_{1}t})$$

while the pseudo-second-order model is given by:

(7) $$q_{t}=\frac{k_{2}q_{e}^{2}t}{1+k_{2}q_{e}t}$$

where $q_{t}$ (mg g^−1^) represents the adsorption capacity at time t, and $k_{1}$ and $k_{2}$ are the rate constants of the pseudo-first-order and pseudo-second-order models, respectively.

The distribution coefficient (K_d_) was calculated using the equation:

(8) $$K_{d}=\frac{q_{e}\times m}{V\times C_{e}}$$

where m is the mass of adsorbent (g) and V is the volume of solution (L). This expression yields a unitless value; however, it represents an apparent distribution coefficient rather than a true thermodynamic equilibrium constant based on activities [41].

The standard Gibbs free energy change was then estimated from:

(9) $$\Delta G^{\circ }=-RTln\;K_{d}$$

It should be noted that experiments were performed at a single temperature (298 K). Therefore, ΔH° and ΔS° could not be determined. Although the conversion $K^{\circ }=K_{L}\times M\times 1000$ is generally preferred in the recent literature, the dimensionless K_d_ calculated here was used as an approximate parameter due to the limitations of the available experimental data. Future studies at multiple temperatures are required for a more rigorous thermodynamic analysis.

Future work will focus on conducting adsorption experiments at multiple temperatures to enable rigorous determination of thermodynamic parameters (ΔH° and ΔS°) using standard methods.

#### 4.7.6. Application to Real Wastewater

A real industrial wastewater sample was collected as a grab sample from the effluent stream of a textile-related facility located in the Rubiki industrial area, 10th of Ramadan City, Egypt. The sample was transported to the laboratory under cooled conditions and stored at 4 °C prior to analysis. To remove coarse suspended solids, the wastewater was filtered through standard filter paper; no chemical pretreatment, dilution, or pH adjustment was performed.

Batch adsorption experiments were conducted under the previously optimized conditions (sorbent dose: 2.5 g L^−1^, contact time: 90 min, temperature: 25 °C, agitation speed: 150 rpm). The wastewater was used without any chemical modification to evaluate the practical applicability of the developed sorbent under real-water conditions. Residual dye concentration was measured by UV–Vis spectrophotometry at λmax, and removal efficiency was calculated accordingly.

## 5. Conclusions

This study reports the successful development of a heteroatom-rich pyridine-based sorbent (**Pyridine PC**) and its CuO-loaded hybrid nanocomposite (**CuO@Pyridine PC**) for the simultaneous removal of malachite green (MG) dye and Cd(II) ions from aqueous systems. The pyridine-based framework provides abundant nitrogen- and oxygen-containing functional groups that act as active adsorption sites, enabling Cd(II) capture mainly through surface complexation, while MG removal occurs via electrostatic attraction, hydrogen bonding, and π–π interactions.

Incorporation of waste-derived CuO nanoparticles creates a synergistic organic–inorganic interface that increases the density and diversity of adsorption sites and significantly improves overall adsorption performance. As a result, **CuO@Pyridine PC** exhibits higher adsorption capacities than pristine **Pyridine PC** for both MG and Cd(II).

The adsorption process follows pseudo-second-order kinetics and is well described by the Freundlich isotherm, indicating heterogeneous adsorption dominated by chemisorption interactions. Thermodynamic analysis confirms that the process is spontaneous and exothermic.

## Figures and Tables

**Table 1:** Kinetic parameters and correlation coefficients for MG and Cd(II) adsorption on **Pyridine PC** and **CuO@Pyridine PC**.

**Adsorbate**	**PFORE**							
	**MG Dye**				**Cd(II)**			
	**q_exp_, mg g^−1^**	**q_e_,** **mg g^−1^**	**K_1_** **(min^−1^)**	**R^2^**	**q_exp_,** **mg g^−1^**	**q_e_,** **mg g^−1^**	**K_1_** **(min^−1^)**	**R^2^**
**Pyridine PC**	34.14	25.9	0.027	0.9698	270.13	121.9	0.047	0.894
**CuO@Pyridine PC**	47.2	26	0.029	0.8955	371.91	198.3	0.05	0.9133
**Adsorbate**	**PSORE**							
	**MG Dye**				**Cd(II)**			
	**q_exp_, mg g^−1^**	**q_e_, mg g^−1^**	**K_2_**·**10^−3^ (g mg^−1^ min^−1^)**	**R^2^**	**q_exp_,** **mg g^−1^**	**q_e_, mg g^−1^**	**K_2_**·**10^−3^ (g mg^−1^ min^−1^)**	**R^2^**
**Pyridine PC**	34.14	37.6	1.85	0.9994	270.13	285.7	0.43	0.9992
**CuO@Pyridine PC**	47.2	50.3	2.13	0.9967	371.91	383.6	0.41	0.9993

**Table 2:** Isotherm parameters and correlation coefficients for MG and Cd(II) adsorption on **Pyridine PC** and **CuO@Pyridine PC** sorbents.

**Adsorbate**	**Langmuir Isotherm**							
	**MG Dye**				**Cd(II)**			
	**q_max,exp_,** **mg g^−1^**	**q_max_, mg g^−1^**	**K_L_, (L mg^−1^)**	**R^2^**	**q_max,exp_,** **mg g^−1^**	**q_max_, mg g^−1^**	**K_L_, (L mg^−1^)**	**R^2^**
**Pyridine PC**	169.8	226	0.11	0.8742	276.5	356	0.08	0.9387
**CuO@Pyridine PC**	176.13	220	0.17	0.8138	368	435	0.15	0.8858
**Adsorbate**	**Freundlich Isotherm**							
	**MG Dye**				**Cd(II)**			
	**K_F_,** **mg** **g^−1^**	**n**		**R^2^**	**K_F_,** **mg** **g^−1^**	**n**		**R^2^**
**Pyridine PC**	21.1	1.36		0.9915	23.9	1.22		0.9987
**CuO@Pyridine PC**	21	1.77		0.9489	53.4	1.39		0.9955
**Adsorbate**	**Temkin Isotherm**							
	**MG Dye**				**Cd(II)**			
	**K_T_, Lg^−1^**	**B_T_,** **J** **mol^−1^**		**R^2^**	**K_T_, Lg^−1^**	**B_T_,** **J** **mol^−1^**		**R^2^**
**Pyridine PC**	1.074	33.6		0.894	2.15	80.03		0.908
**CuO@Pyridine PC**	2.055	43.38		0.8941	1.06	95.04		0.9358

**Table 3:** Langmuir and Freundlich isotherm parameters obtained from nonlinear regression for the adsorption of MG and Cd(II) adsorption on **Pyridine PC** and **CuO@Pyridine PC** sorbents.

**Adsorbate**	**Langmuir Isotherm**							
	**MG Dye**				**Cd(II)**			
	**q_max,exp_,** **mg g^−1^**	**q_max_, mg g^−1^**	**K_L_, (L mg^−1^)**	**R^2^**	**q_max,exp_,** **mg g^−1^**	**q_max_, mg g^−1^**	**K_L_, (L mg^−1^)**	**R^2^**
**Pyridine PC**	169.8	368.5	0.02	0.999	276.5	679	0.033	0.9996
**CuO@Pyridine PC**	176.13	380.9	0.03	0.9886	368	771.2	0.05	0.9956
**Adsorbate**	**Freundlich Isotherm**							
	**MG Dye**				**Cd(II)**			
	**K_F_,** **mg** **g^−1^**	**n**		**R^2^**	**K_F_,** **mg** **g^−1^**	**n**		**R^2^**
**Pyridine PC**	10.8	1.27		0.993	25.9	1.41		0.9985
**CuO@Pyridine PC**	15.67	1.42		0.9951	35.67	1.42		0.9952

**Table 4:** Thermodynamic parameters for MG and Cd(II) adsorption on **Pyridine PC** and **CuO@Pyridine PC**.

Adsorbent	T, (K)	∆H°, (kJ/mol)	∆S°, (J/mol·K)	∆G°, (kJ/mol)	R^2^
**Pyridine PC (MG)**	298	−25.59	−67.48	−5.48	0.9967
	308			−4.81	
	318			−4.13	
	328			−3.46	
**CuO@Pyridine PC** **(MG)**	298	−52.93	−149.79	−4.46	0.9798
	308			−8.29	
	318			−5.3	
	328			−3.8	
**pyridine PC (Cd(II))**	298	−37.98	−99.08	−6.73	0.9718
	308			−5.68	
	318			−4.64	
	328			−3.59	
**CuO@Pyridine PC** **(** **Cd(II))**	298	−56.12	−150.38	−8.76	0.9811
	308			−7.17	
	318			−5.58	
	328			−3.99	

**Table 5:** Desorption efficiency of Cd(II) and MG over five consecutive adsorption–desorption cycles.

Cycle Number	Cd(II) Desorption Efficiency (%) ^1^		MG Desorption Efficiency (%) ^1^	
	Pyridine PC	CuO@pyridine PC	Pyridine PC	CuO@pyridine PC
**1**	94.3	97.5	90.2	96.9
**2**	90.24	94.6	87.6	94.4
**3**	87.96	92.84	83.5	91.53
**4**	83.24	91.67	82.72	90.98
**5**	80.98	90.05	81.14	89.74

^1^ Desorption was carried out using 0.1 M HCl for Cd(II) and 0.2 M NaOH for MG.

**Table 6:** Comparative adsorption capacities for MG dye and Cd(II) ions.

Adsorbent	Pollutant	q_max_ (mg g^−1^)	Reference
Citric acid-cross-linked β-cyclodextrin matrix	MG dye	~904 mg g^−1^	[31]
Functional polymer (TPA-POP)	MG dye	~756 mg g^−1^	[32]
Nano-bentonite	MG dye	~98.6% removal efficiency (~variable)	[33]
Activated carbon from coconut shell	Cd(II)	~33.7 mg g^−1^	[34]
Alkali-activated fly ash/metakaolin adsorbents	Cd(II)	8.5–72.8 mg g^−1^	[35]
Synthetic Na-X zeolite	Cd(II)	~185–268 mg g^−1^	[36]
inorganic porous nanocomposites (Iron oxide)	Cd(II)	4.1 mg g^−1^	[37]
CAN-zeolite	Cd(II)	68 mg g^−1^	[38]
Pyridine PC (this work)	MG dye	169.8 mg g^−1^	Current study
CuO@Pyridine PC (this work)	MG dye	176.13 mg g^−1^	Current study
Pyridine PC (this work)	Cd(II)	276.5 mg g^−1^	Current study
CuO@Pyridine PC (this work)	Cd(II)	368 mg g^−1^	Current study

## Data Availability

The data supporting the findings of this study are available from the corresponding author upon reasonable request.

## Supplementary Materials

The following supporting information can be downloaded at: https://www.mdpi.com/article/10.3390/molecules31091501/s1, Figure S1: ^1^H NMR spectrum of the **Pyridine PC** sorbent; Figure S2: ^13^C NMR spectrum of the **Pyridine PC** sorbent; Figure S3: Mass spectrum of the **Pyridine PC** sorbent; Figure S4: Pore size distribution curves of (a) Pyridine-PC and (b) CuO@Pyridine-PC derived from nitrogen adsorption–desorption analysis; Figure S5: Zeta potential profile of the **CuO@Pyridine PC** composite; Figure S6: EDX elemental mapping of C, N, and O in **CuO@Pyridine PC** Before and after adsorption of Cd(II). Scale bar: 100 µm; Figure S7: Determination of pH_PZC_ of **Pyridine PC** and **CuO@Pyridine PC** composite (SD: 2 g L^−1^; background electrolyte: 0.1 M NaCl; agitation time: 24 h; agitation speed: 200 rpm; T: 25 °C); Figure S8: Nonlinear Langmuir isotherm fitting for MG and Cd(II) adsorption **Pyridine PC** and **CuO@Pyridine PC**. (a) **Pyridine PC**–MG, (b) **CuO@Pyridine PC**–MG, (c) **Pyridine PC**–Cd(II), (d) **CuO@Pyridine PC**–Cd(II); Figure S9: Nonlinear Freundlich isotherm fitting for MG and Cd(II) adsorption **Pyridine PC** and **CuO@Pyridine PC**. (a) **Pyridine PC**–MG, (b) **CuO@Pyridine PC**, (c) **Pyridine PC**–Cd(II), (d) **CuO@Pyridine PC**–Cd(II); Figure S10: Van’t Hoff plots for (a) MG dye and (b) Cd^2+^ adsorption on **Pyridine PC** and **CuO@Pyridine PC.** Figure S11. Van’t Hoff plots for (a) MG dye and (b) Cd^2+^ adsorption on **Pyridine PC** and **CuO@Pyridine PC**. Table S1. Kinetic, isotherm, and thermodynamic equations for MG dye and Cd(II) adsorption.

## Abbreviations

The following abbreviations are used in this manuscript:

**Pyridine PC**	heteroatom-rich pyridine-based adsorbent
MG	Malachite Green
Cd(II)	Cadmium(II) ion
R^2^	Correlation coefficient
PFORE	Pseudo-first-order equation
PSORE	Pseudo-second-order equation
pHpzc	pH at point of zero charge
qe	Equilibrium adsorption capacity
