# Supplementary material for: CuO@Pyridine Composite for Efficient Removal of Malachite Green and Cd(II) from Water: Adsorption Performance and Mechanistic Insights
Source: Molecules. 2026 Apr 30;31(9):1501. doi: 10.3390/molecules31091501 (PMC13164746; doi:10.3390/molecules31091501)
Supplement: Supplementary file 1 [file molecules-31-01501-s001.zip › molecules-4250731-supplementary.pdf]

CuO@Pyridine Composite for Efficient Removal of Malachite Green and Cd(II) from Water: Adsorption Performance and Mechanistic Insights

Marwa M. Abdeen[1], Mohamed G. Abouelenein[2*], Marwa Abd Elfattah[1], Safinaz H. El-Demerdash[2], Marwa A. Abdelhameed[2], Sara M. Elnagar[2], Mariam T. yasin[2], Donia F. Elhadad[2], Mohamed Mostafa A. Mohamed[3*]

Supplementary data

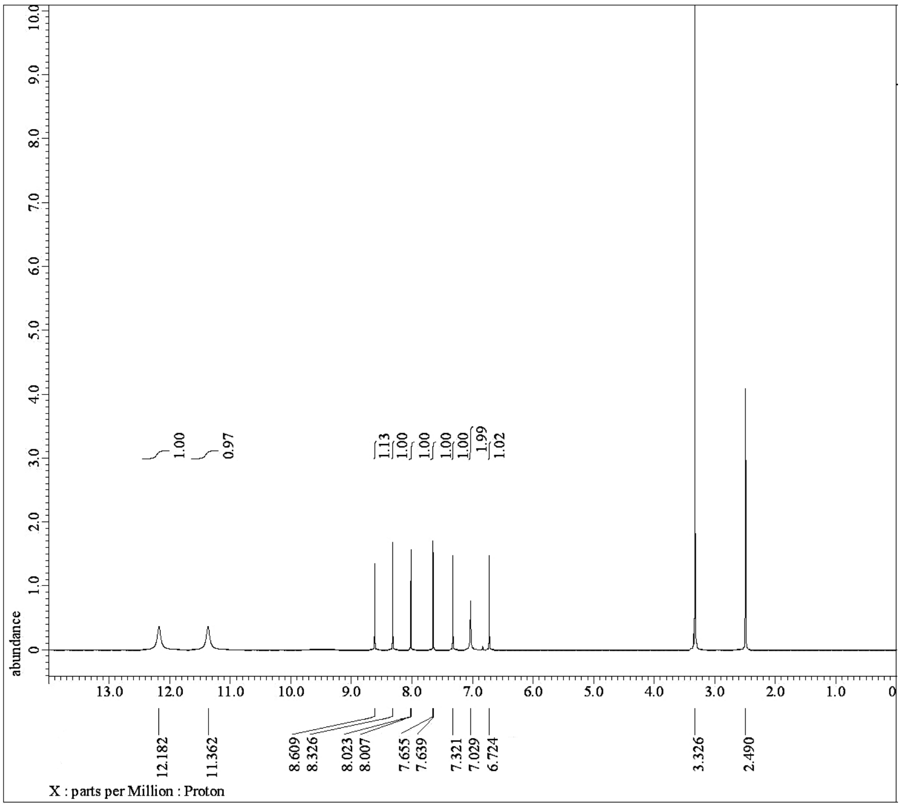

Figure S1. ^{1}H NMR spectrum of Sorbent **Pyridine PC**.

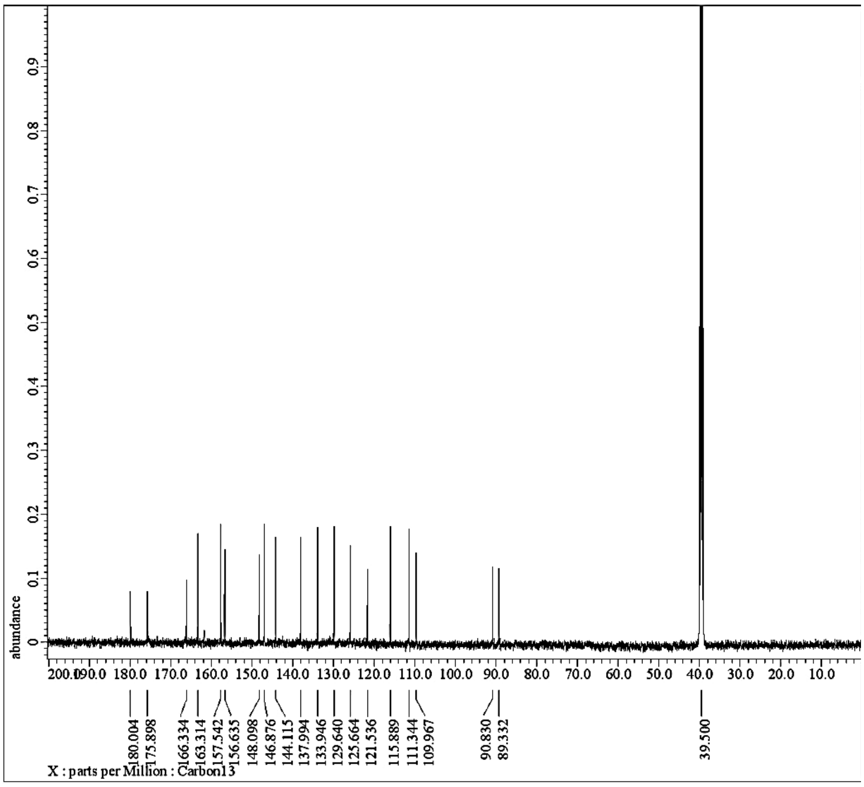

Figure S2. ^{13}C NMR spectrum of Sorbent **Pyridine PC**.

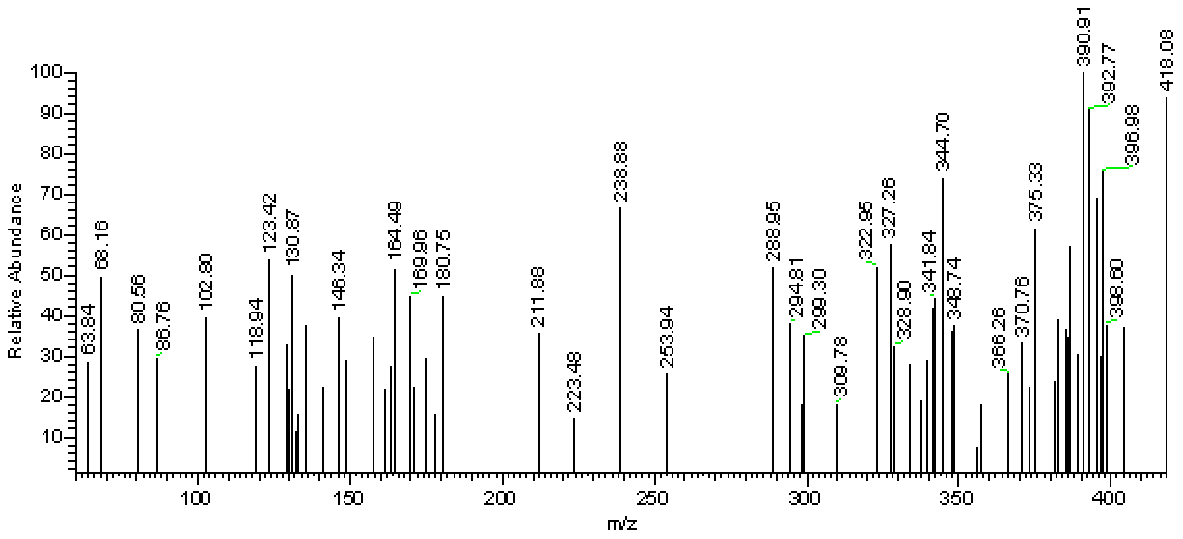

Figure S3. Mass spectrum of Sorbent **Pyridine PC**.

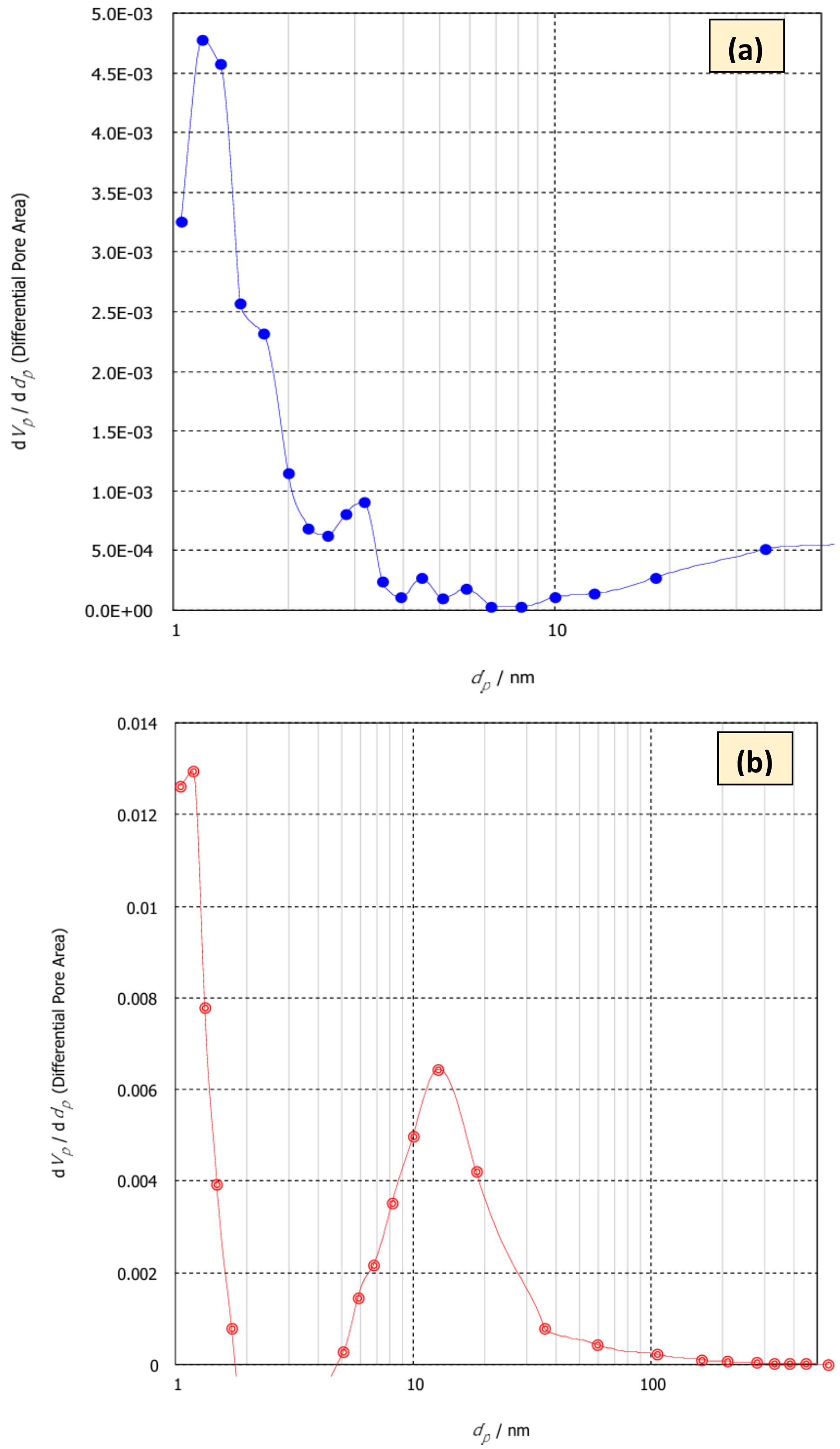

Figure S4. Pore size distribution curves of (**a**) **Pyridine-PC** and (**b**) **CuO@Pyridine-PC** derived from nitrogen adsorption–desorption analysis.

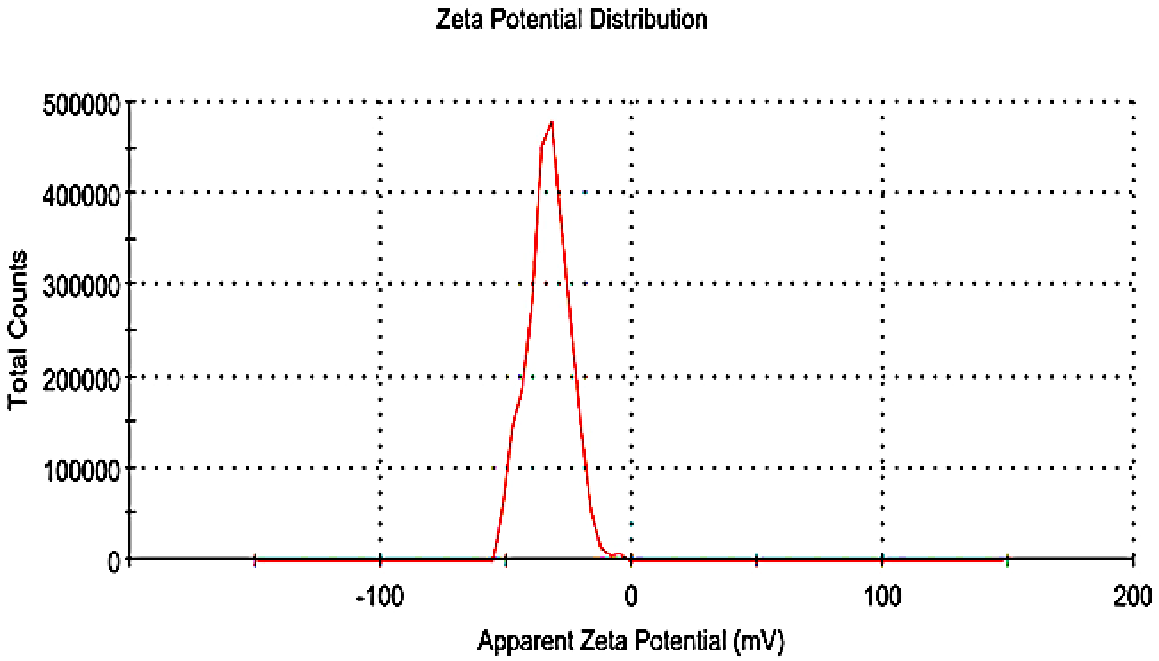

Figure S5. Zeta potential profile of **CuO@Pyridine PC** composite.

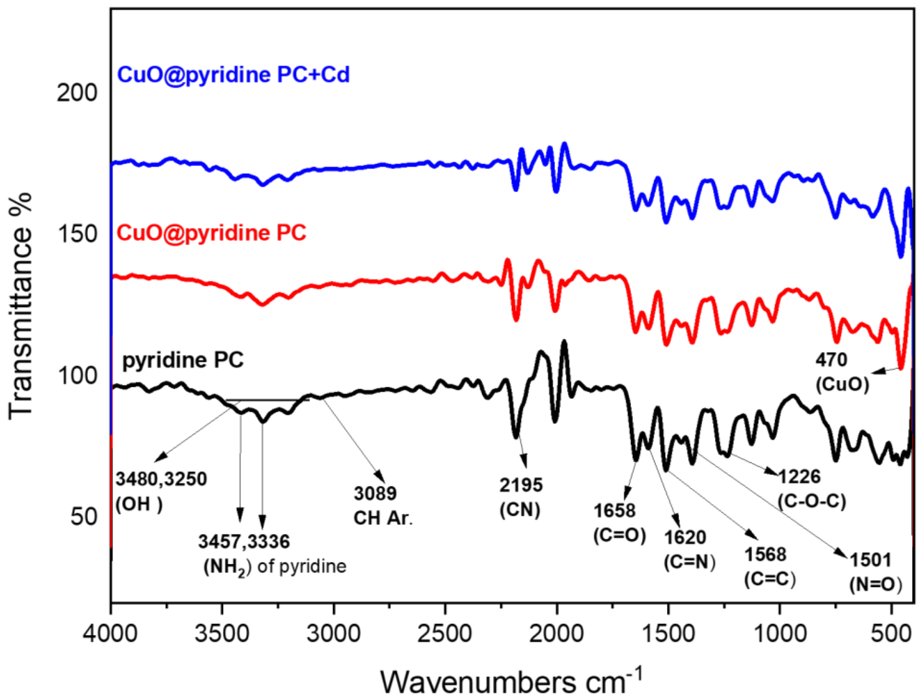

Figure S6. Comparative FT-IR spectra of Pyridine PC and CuO@Pyridine PC before and after five adsorption–desorption cycles.

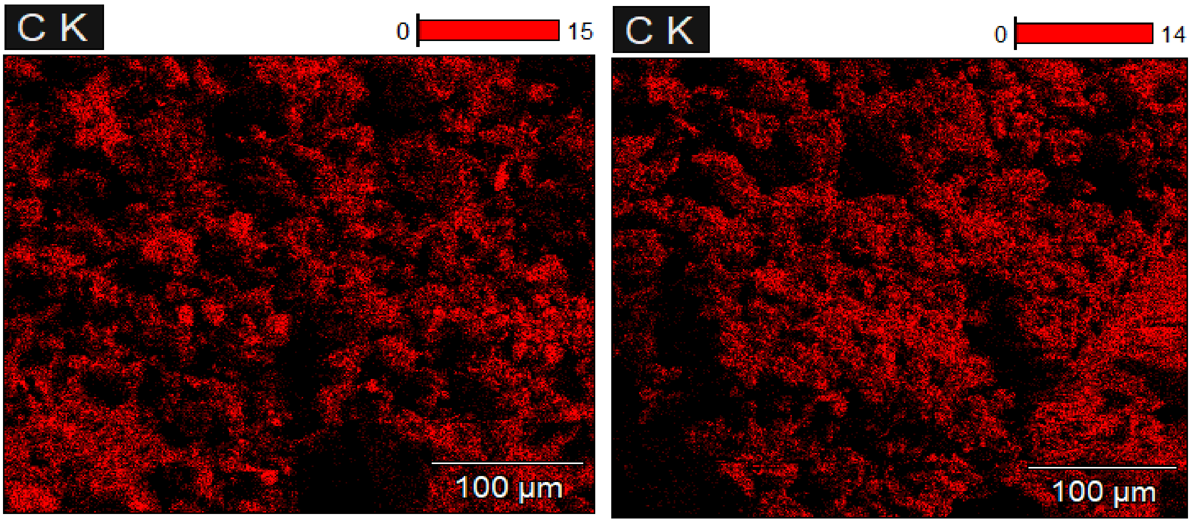

CuO@Pyridine PC   **CuO@Pyridine PC + Cd(II)**

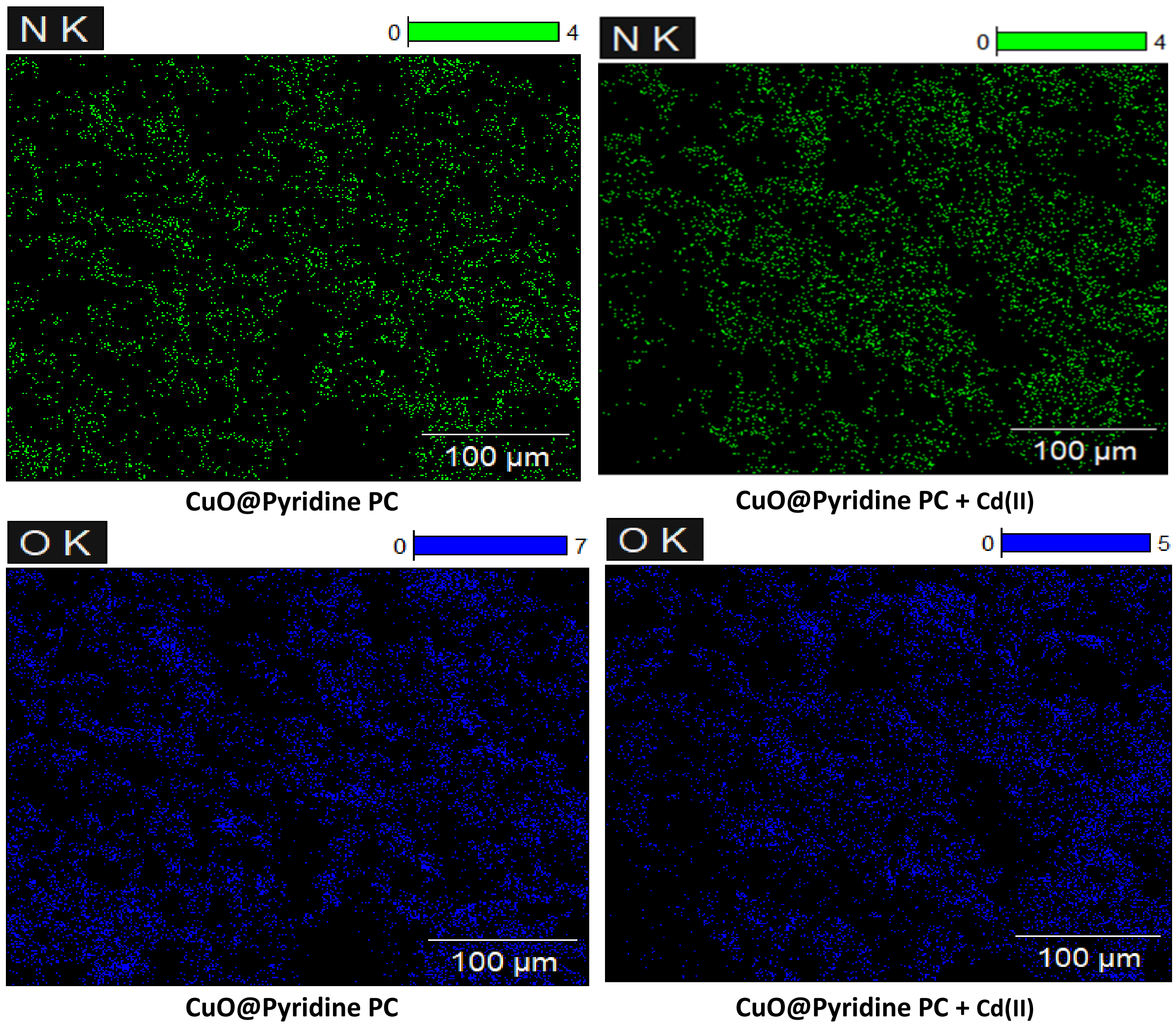

Figure S7. EDX elemental mapping of C, N, and O in **CuO@Pyridine PC** Before and after adsorption of Cd(II). Scale bar: 100 µm.

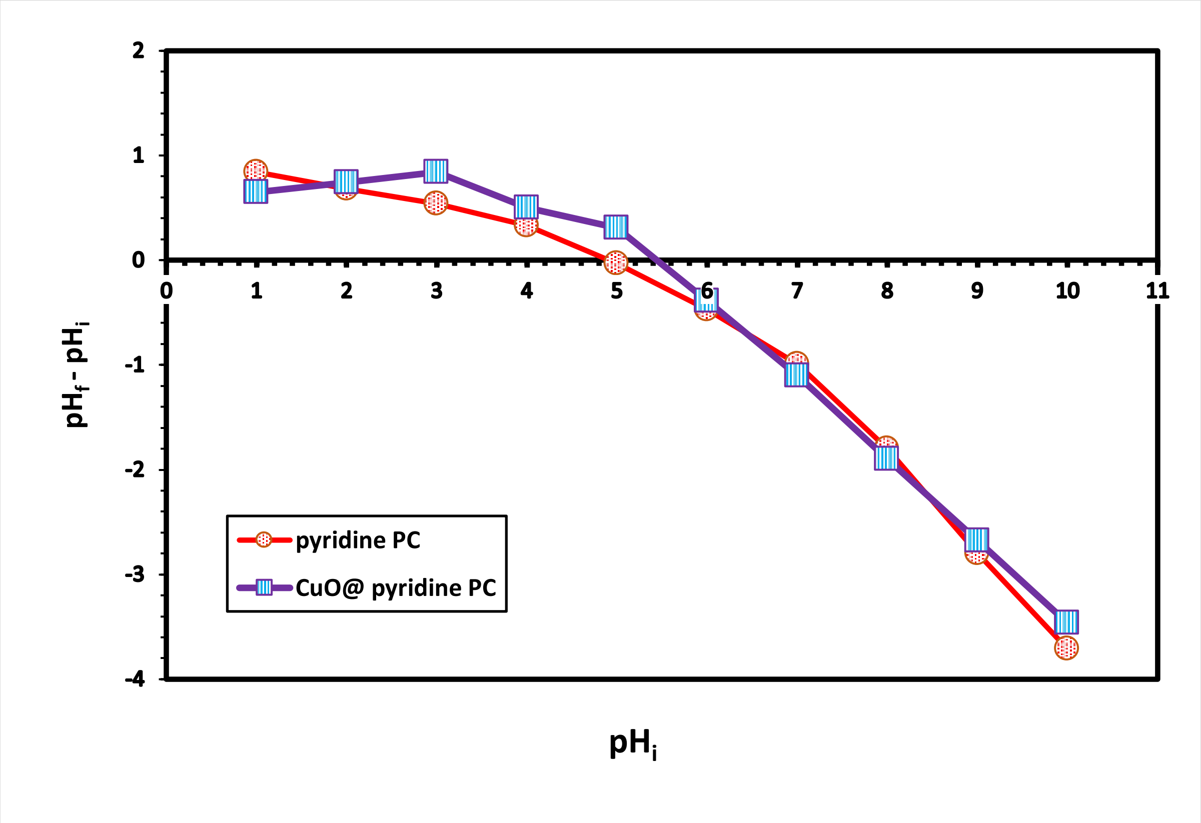

Figure S8. Determination of pH_{PZC} for by **Pyridine PC** and **CuO@Pyridine PC** composite. (SD: 2 g L−1; Background salt: NaCl (0.1 M); agitation time: 24 h; agitation speed: 200 rpm; T: 25 °C).

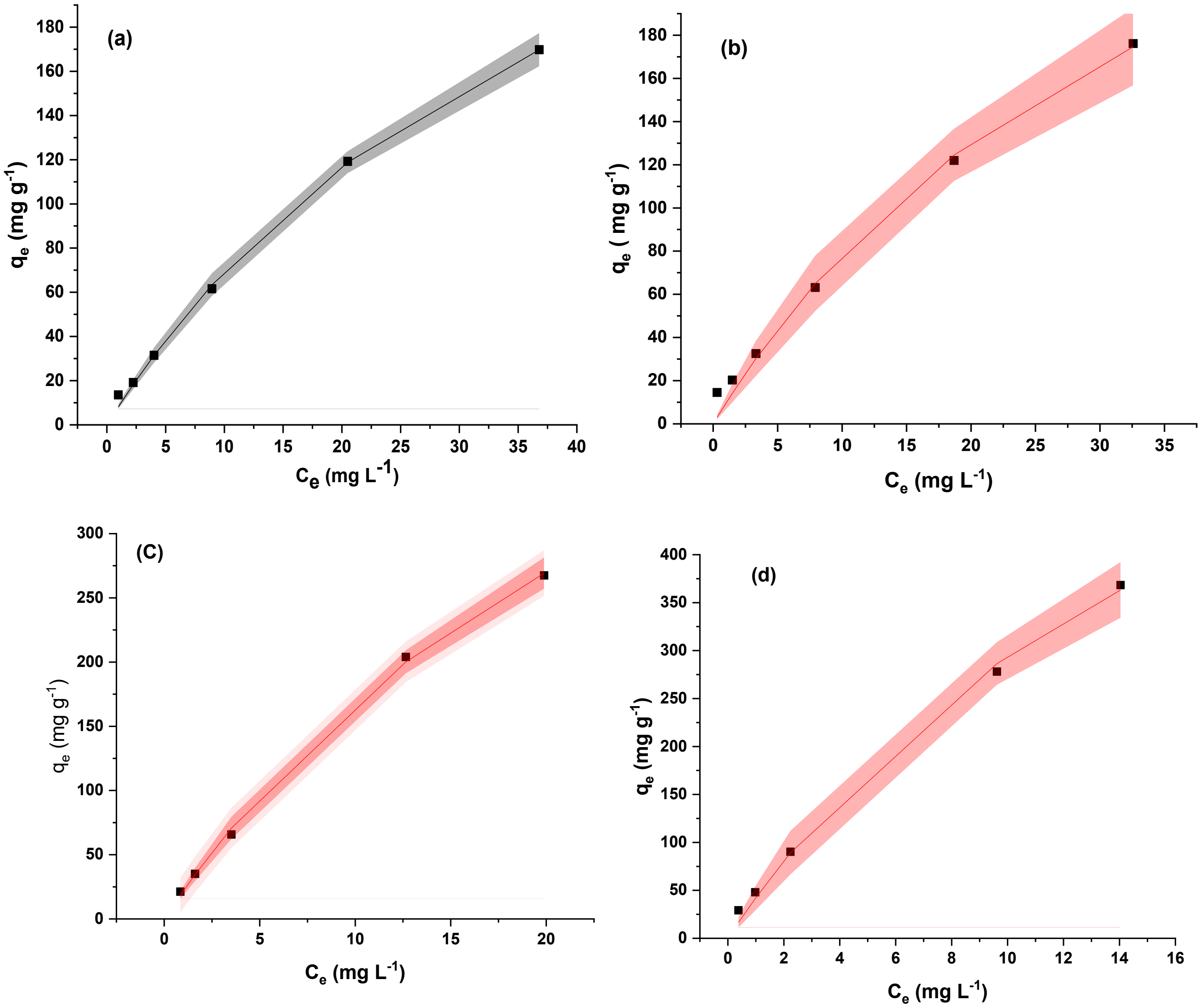

Figure S9. Nonlinear Langmuir isotherm fitting for MG and Cd(II) adsorption **Pyridine PC** and **CuO@Pyridine PC**. (**a**) Pyridine PC–MG, (**b**) CuO@Pyridine PC–MG, (**c**) Pyridine PC–Cd(II), (**d**) CuO@Pyridine PC–Cd(II).

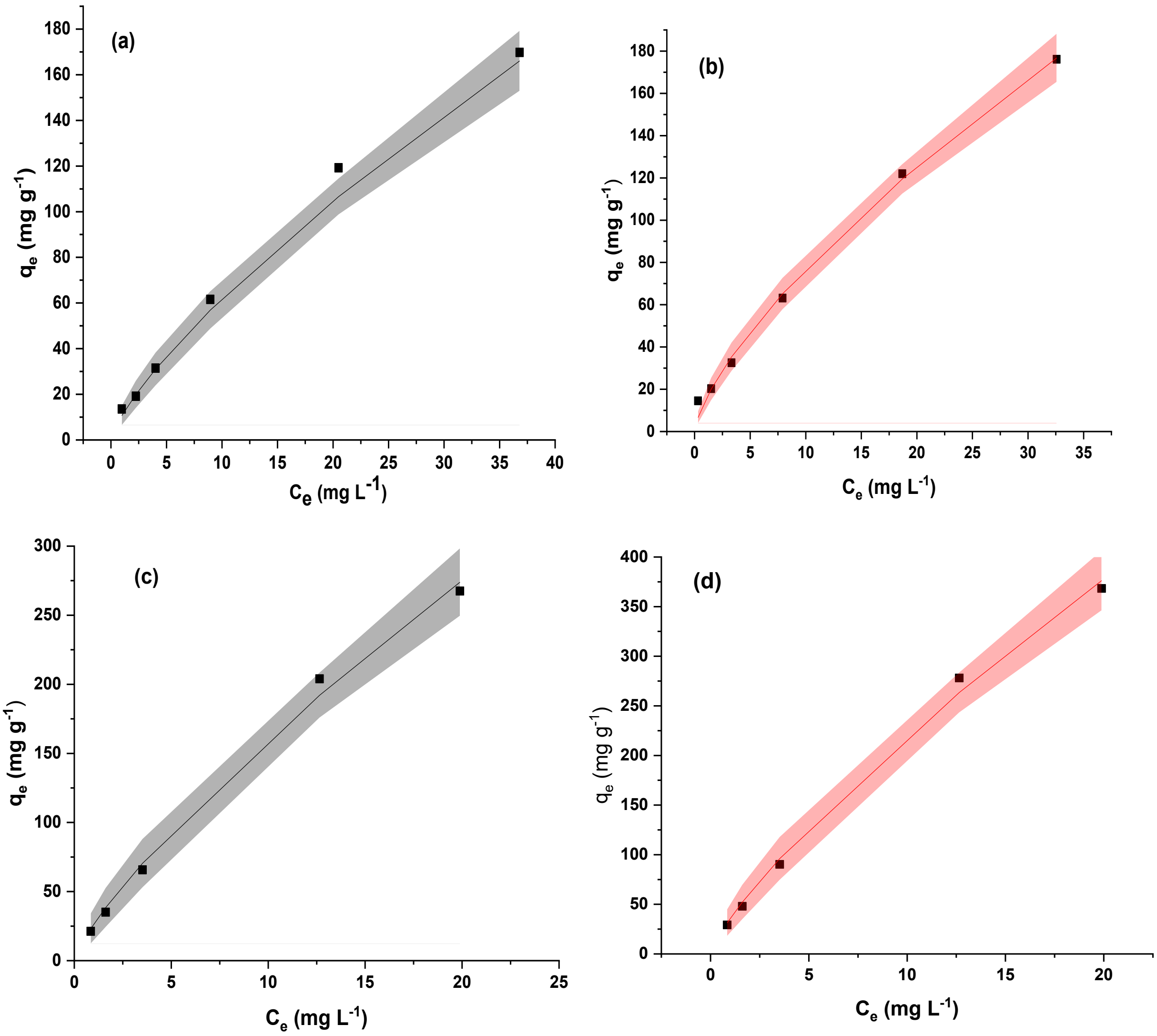

Figure S10. Nonlinear Freundlich isotherm fitting for MG and Cd(II) adsorption **Pyridine PC** and **CuO@Pyridine PC**. (**a**) Pyridine PC–MG, (**b**) CuO@Pyridine PC–, (**c**) Pyridine PC–Cd(II), (**d**) CuO@Pyridine PC–Cd(II).

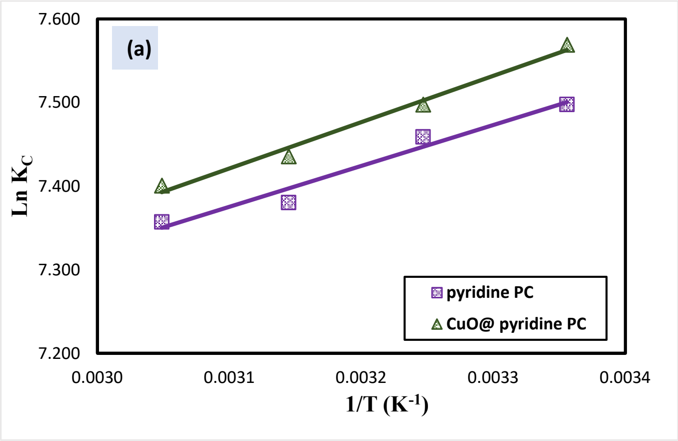

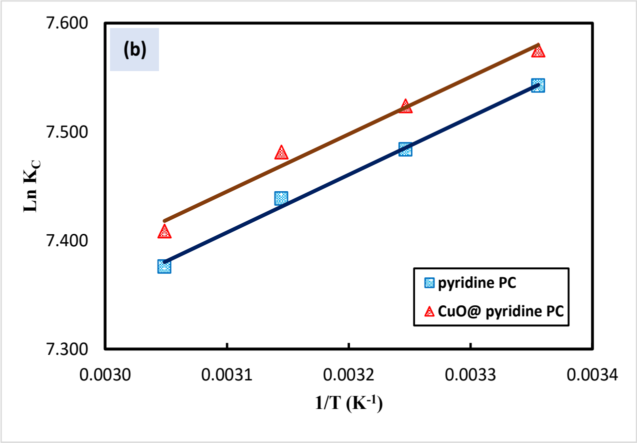

Figure S11. Van't Hoff plots for (**a**) MG dye and (**b**) Cd^{2+} adsorption on **Pyridine PC** and **CuO@Pyridine PC**.

Table S1. Kinetic, isotherm, and thermodynamics equations for MG dye and Cd(II) adsorption process.

**Kinetics**	**Equations**
**Pseudo-first-order**	$\log(q_e - q_t) = log\, q_e - \left(\frac{K_1}{2.303}\right) t$
**Pseudo-second-order**	$\frac{t}{q_t} = \frac{1}{K_2\, q_e^2} + \frac{1}{q_e}\, t$
**Isotherms**	**Equations**
**Langmuir model**	$\frac{C_e}{q_e} = \frac{C_e}{q_{max}} + \frac{1}{q_{max}\, K_L}$
**Freundlich model**	$\log(q_e) = \log K_f + \frac{1}{n} \log C_e$
**Temkin model**	$q_e = \frac{RT}{b_T} ln\, K_T C_e$
**Thermodynamics**	**Equations**
$\ln K = \frac{-\Delta H^o}{RT} + \frac{\Delta S^o}{R}$	
$\Delta G = \Delta H - T\Delta S$	
